# *Cadê o Kauê?* Co‐design and acceptability testing of a chat‐story aimed at enhancing youth participation in the promotion of mental health in Brazil

**DOI:** 10.1111/jcpp.14078

**Published:** 2024-12-20

**Authors:** Gabriela Pavarini, Sheila Giardini Murta, Josimar Antônio de Alcântara Mendes, Felipe Rodrigues Siston, Rafa Ribeiro Alves de Souza, Rafaela de Oliveira da Cunha, Julyana Alves Ferreira, Victor Hugo de Lima de Santos, Brenda Thallys Rocha Seabra, Talk2U, Nicolás Ferrario, Lu Suarez Battan, Bia Matera, Iasmin Coni, Lucca Rosa Pereira, Thayna Iglesias, Ana Luiza Savi, Daniel Foscarini, Kathia Calil, Felipe Corvello, Sômolo Silvestre Salvador, Amparo Guindón, Silvia Lordello, Larissa Polejack, Alice Christovam Constantino, Alicia Mendes, Ana Clara Freitas Ferreira, Ana Vitória Oliveira da Silva, Brenda lorrane Ferreira Feitosa, Alves, Emerson Gomes dos Santos, Hevellyn Sofia Elvino Pinheiro, Iasmyn Brito dos Santos, Isis Santos Goulart, Jamili Maine Lima Campos, Maria Layza Paixão, Milena Sousa, Mutula Manuel Simão João, Natália Samuel Pitcella Borba, Raissa Barbosa da Silva, Raissa Amarante, Yasmin Bilek, Yasmin Flores da Silva, Gabriela Staerke de Rezende, Jonas de Oliveira Bertucci, Claudia Cavalcante de Carvalho Weber, Janete Araújo da Silva, Rodrigo Araújo Magalhães, Rosimeire Aguiar Pereira Lopes, Mikael Silva Rocha, Ana Paula Santiago S. Andrade, Fernanda Bernardes Silveira Costa, Maria Salete Guerra Maranhão Bezerra, Gina Vieira Ponte, Thiago Freire, Tony Marcelo Gomes de Oliveira, Ilina Singh

**Affiliations:** ^1^ Ethox Centre, Oxford Population Health University of Oxford Oxford UK; ^2^ Department of Social Policy and Intervention University of Oxford Oxford UK; ^3^ Department of Clinical Psychology, Institute of Psychology University of Brasília Brasília Brazil; ^4^ Responsible Technology Institute, Department of Computer Science University of Oxford Oxford UK; ^5^ Engajadamente Youth Collaborative Group Brasília Brazil; ^6^ Talk2U, Health‐Tech Company São Paulo Brazil; ^7^ Department of Psychiatry University of Oxford Oxford UK

**Keywords:** youth participation, video games, storytelling, digital interventions, adolescents, user‐centred design, civic engagement, co‐design, mental health, wellbeing, co‐production, empowerment, peer support, gaming, chatbot

## Abstract

**Background:**

Adolescent mental health is vital for public health, yet many interventions fail to recognise adolescents as proactive community contributors. This paper discusses the co‐design and acceptability testing of a chat‐story intervention to enhance Brazilian adolescents' participation in the promotion of mental health in their peer communities. We specifically highlight the iterative process of co‐creating this intervention with community stakeholders.

**Methods:**

The co‐design was led by researchers, a youth collaborative group, and health‐tech experts. Part 1 included quantitative (*n* = 1,768) and qualitative (*n* = 46) studies with Brazilian adolescents aged 15–18 for priority‐setting. Part 2 involved co‐creation and technical production, with input from youth advisors (*n* = 24), school staff (*n* = 11), and policy experts (*n* = 3). In Part 3, the chat‐story was user tested (*n* = 32). Parts 4 and 5 assessed acceptability through a qualitative study in schools (*n* = 138) and initial efficacy during an online campaign (*n* = 795).

**Results:**

Participants aspired to support their peers' mental health in schools, both one‐to‐one and collectively, but felt unprepared. This informed the chat‐story's goal of enhancing peer support and collective action skills. Themes identified during Part 1, such as prejudice and academic pressure, were woven into the narrative to raise awareness of the social determinants of mental health, drawing from real‐life stories. In the final story, players search for their missing best friend at school, uncovering his anxiety struggles and practicing skills such as empathic listening and partnership building. A manual for teachers was collaboratively designed for use within school settings, supplementing direct‐to‐user online applications. Acceptability testing showed participants found the tool authentic and user‐friendly. Online users perceived the tool as preparing and motivating them to offer peer support and engage in collective action.

**Conclusions:**

The immersive co‐creation model, enriched by input from key stakeholders, yielded a relevant and well‐received intervention for Brazilian adolescents. Co‐designed creative tools like chat‐stories hold promise as digital mental health tools, fostering awareness, critical reflection, and inspiring adolescents to drive positive social change.

## Introduction

The United Nations emphasises the importance of involving young people in decisions that concern them, calling them ‘assets of society’ and ‘important agents of change’ (United Nations, 2007). Yet, health and well‐being initiatives traditionally frame young people as passive ‘recipients’ of resources and interventions, rather than active agents in promoting community well‐being. In a recent mapping of adolescent health research, we found that only 1% of studies adopted participatory practices (Sellars, Pavarini, Michelson, Creswell, & Fazel, 2021), and similar dynamics apply to services and policy development (Clark et al., 2020). This represents a critical missed opportunity. Youth participation can enhance the reach, relevance and effectiveness of mental health initiatives, and offer innovative solutions to young people's mental health problems (Pavarini, Lorimer, Manzini, Goundrey‐Smith, & Singh, 2019; Reed et al., 2021). Participation is not only a key priority for young people themselves (Patton et al., 2016) but it is also in young people's best interests (UNICEF, 1989), as well‐being initiatives can have a direct impact on their lives, futures and welfare.

One promising way in which young people's potential can be harnessed is through co‐designing interventions (Gatera & Pavarini, 2020; Pavarini, Reardon, Mawdsley et al., 2024). Co‐design is a process whereby researchers, designers and developers partner up with other stakeholders across the entire process to co‐develop an intervention (Sanders & Stappers, 2008). When co‐design includes young people, it helps ensure relevant, youth‐friendly interventions that address young people's needs and take account of their preferences, while employing scientific rigour and a theoretical grounding (Bevan Jones et al., 2020).

Over the past decade, interest in involving young people in mental health interventions, particularly digital ones, has increased (Bevan Jones et al., 2020), in efforts to overcome the attrition and low adherence that commonly characterise these interventions (Hollis et al., 2017; Scholten & Granic, 2019). Young people's participation is typically consultative, implemented across a number of consultation meetings with a youth group, rather than co‐led by young people. Furthermore, most co‐designed digital interventions have been developed in high‐income settings, including Australasia, North America and Europe (Bevan Jones et al., 2020). However, more digital resources are needed to help address the healthcare gaps in low‐ and middle‐income countries (LMICs) and reduce the stigma associated with poor mental health (Carter, Araya, Anjur, Deng, & Naslund, 2021; Naslund & Deng, 2021).

Digital interventions offer unprecedented opportunities for young people in Brazil, where smartphone access exceeds 91% amongst those aged 15–17 years, and over 96% of that age group access the internet regularly (TIC, 2021). Brazil is one of the countries that was worst affected by the COVID‐19 pandemic (Sott, Bender, & da Silva Baum, 2022), and Brazilian adolescents over 15 years of age reported the greatest emotional difficulties of all age groups during this period (Barros et al., 2022). With most Brazilians perceiving mental health as the top health concern facing the country today (IPSOS, 2023), there is significant scope for mental health interventions grounded on youth participation.

In this project, we aimed to co‐design a digital intervention for and with Brazilian adolescents, which not only had youth participation at the heart of its design process, but also as its target outcome. Rather than directly alleviating adolescents' symptoms or improving their mental state, the intervention focused on strengthening young people's participation in promoting mental health and well‐being in their communities. Youth empowerment can itself promote young people's mental health and well‐being (Ballard & Syme, 2016), and many researchers have advocated encouraging young people to take an active role – not only in shaping their own mental health, but also in championing well‐being initiatives (e.g., awareness‐raising campaigns or peer‐led support groups) (Clark et al., 2020; Gatera & Pavarini, 2020; Vijayaraghavan et al., 2022).

Our digital intervention took the form of a “story‐telling chatbot”, or a *chat‐story*. This is a type of virtual experience, pioneered by the health‐tech company Talk2U, in which a narrative unfolds as players interact with fictional, automated character(s) (bots) via text messaging (Talk2U, 2022). The characters share accounts of their ‘real‐time’ experiences in the form of text, audio, photographs and videos, and players drive the narrative forward by selecting or writing responses. Chat‐stories are a gamified variation of ‘digital storytelling’, a method that has been used to help people make sense of their experiences by engaging with the experiences of others (De Vecchi, Kenny, Dickson‐Swift, & Kidd, 2016). Like other arts‐based interventions (Fancourt, 2017), the emotionally engaging nature of this type of resource makes it a promising tool to engage and inspire young people.

### Aim of the study

This study aimed to co‐design and evaluate the acceptability of a digital intervention – a chat‐story – to increase youth participation in the promotion of mental health in Brazil. We followed an immersive co‐design process, working in a core team of researchers, adolescents and creative industry experts, supported by a larger network of adolescents, school staff, and experts in policy, technology and audio‐visual design. The project encompassed (1) a mapping phase to gather young people's aspirations regarding their own possible participation in the promotion of mental health and well‐being; (2) co‐creation (design and refinement of the intervention); (3) user‐testing; (4) a qualitative acceptability study; and (5) an online dissemination campaign that gathered further acceptability data and initial efficacy results.

## Overview of the co‐design process

**Figure 1:**
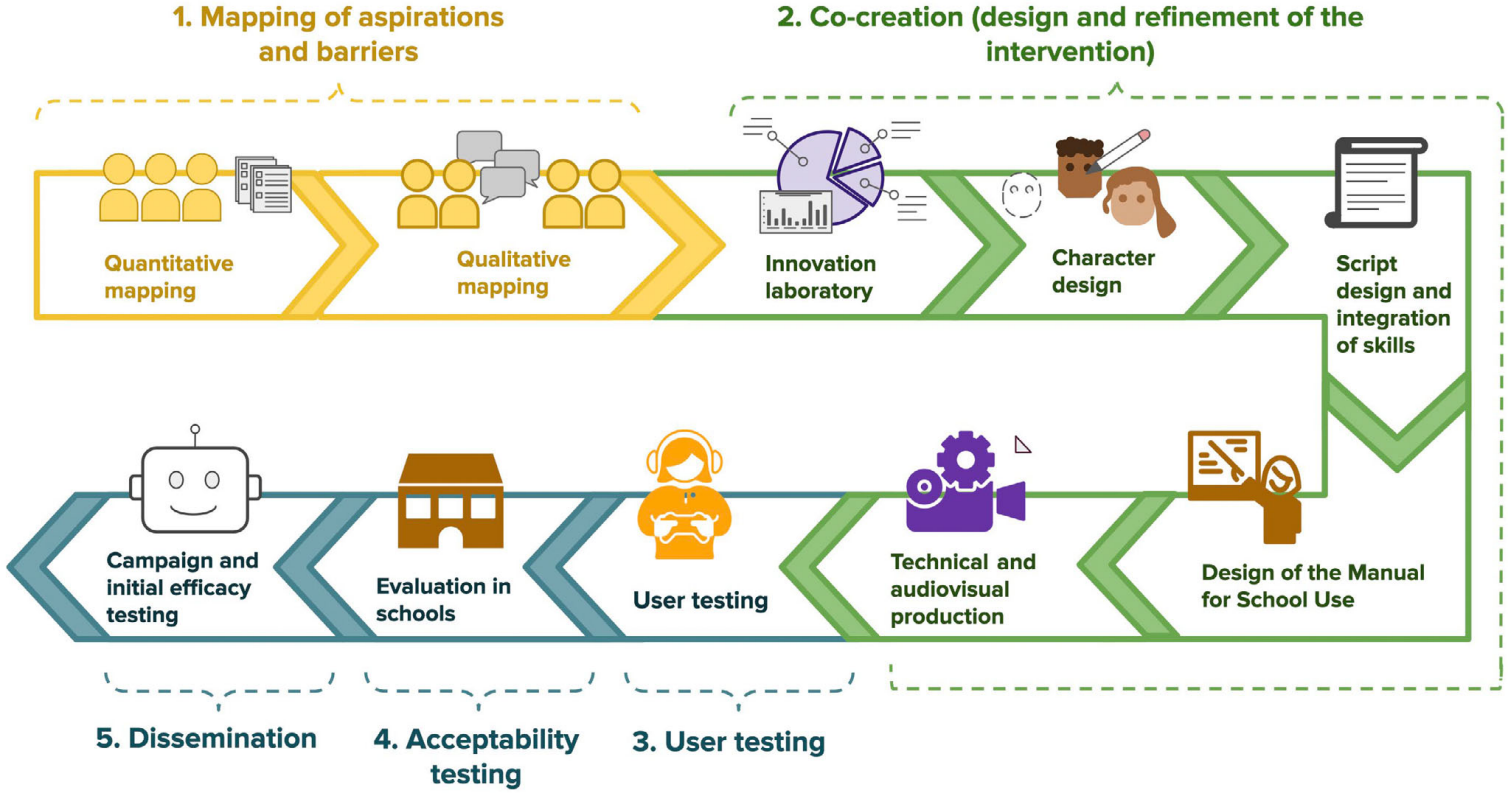
Overview of co‐design process

Our co‐design process is depicted in Figure 1 and detailed in the sections below. The core co‐design team consisted of the following: four researchers based at the universities of Oxford and Brasília with expertise in psychology, public health, communication and ethics (GP, SM, JM, and FS); a youth collaborative group composed of five young people aged 17–20 years (at the time of recruitment) based in Brasilia with lived experience of community engagement and/or mental health difficulties and interest in the creative arts (RS, JF, RC, VS, and BS); and three creative experts specialising in storytelling and scriptwriting (NF, BM and IC from Talk2U). Following the UK National Institute for Healthcare Research guideline on co‐producing a research project (NIHR, 2018), this team worked in a non‐hierarchical manner, sharing power and responsibility throughout, and particularly in the co‐creation phase (Part 2). Youth collaborators acted as peer researchers and were remunerated monthly, with research training and mentorship provided throughout the project.

Input from advisory committees and experts in public policy (Part 2c), as well as two rounds of user testing (Part 3) and the acceptability study (Part 4) helped shape and refine the chat‐story. The mapping phase, as well as stakeholder involvement and user testing, was co‐led by the researchers and the collaborative youth group. The acceptability study was led by the researchers with support from the youth group. Health‐tech partners collaborated in the co‐creation and led the audio‐visual and technical development and the campaign (Part 5). More information on our co‐design approach is provided by Siston et al. (2023) and given in the project's video introduction at https://www.youtube.com/watch?v=XKBigTp8h3w.

### Ethics considerations

The project received ethics approval from the Oxford Tropical Research Ethics Committee (Ref. 501‐21, Ref. 550‐21), the University of Brasilia Humanities and Social Sciences Ethics Committee (Ref. 4.688.652, Ref. 5.196.133) and the Brazilian National Ethics Commission (Ref. 4.932.068, Ref. 5.510.769). Informed consent was gained from all participants or their carers (with young people's assent) for each stage of the project.

## Part 1. Mapping phase

The mapping phase consisted of two studies aimed at identifying the aspirations of and barriers faced by young people in Brazil regarding the promotion of mental health and wellbeing amongst their peers. The second study additionally investigated young people's perceptions of relevant challenges to youth mental health in Brazil.

### Quantitative mapping

We conducted secondary analysis of data from a global survey study disseminated via UNICEF's U‐Report platform (for full detail, see Pavarini et al., 2023). The survey included multiple‐choice questions on participants' aspirations concerning their possible participation in promoting mental health amongst Brazilian adolescents, their preferred settings, and the barriers they felt they would face. Our analysis included all participants who resided in Brazil and were aged 15–18 years. The sample comprised 1,768 participants across the five regions of Brazil. With regards to gender, 60.2% identified as a girl/woman, 39.1% as a boy/man, 0.3% chose ‘other gender’, and 0.4% did not report. Table 1 shows the percentage of participants endorsing each response option. Participants' main aspirations for involvement were joining a mental health project, improving the support available to young people and providing peer support. Most participants chose ‘school’ as the setting where they could make the most difference to the mental health of others. The main barriers to meaningful participation included a lack of information on mental health and how to get involved, and low self‐efficacy.

**Table 1:** Aspirations, spheres of influence and barriers to participation in promoting mental health amongst Brazilian adolescents

Domain	Percentage of participants
Aspirations (*n* = 1,768)	
**Be part of a mental health project/campaign**	**28.96**
**Help improve the support available**	**24.32**
**Support each other**	**22.00**
Help collect information on mental health	13.29
Inform local leaders about challenges	10.52
Other	0.90
**Spheres of influence** (*n* = 1,676)	
**School**	**52.57**
Community spaces	22.20
Online	9.84
Home	8.29
Work	1.79
Other	5.31
**Barriers to participation** (*n* = 1,522)	
**Not enough information**	**29.89**
**Unsure if I can make a difference**	**26.02**
**Don**'**t know how to get involved**	**23.72**
Not a priority for young people	20.30
Other	0.07

Questions were single select multiple choice. Responses that informed priority setting for chat‐story are in bold.

### Qualitative mapping

As the school was a critical sphere of influence, a qualitative study assessed young people's perceived barriers to participation in promoting mental health in schools and their aspirations for involvement. The results from this study are reported in Mendes et al. (2024). Multiple barriers to participation were identified, including feeling unprepared to talk about mental health and/or support others, reluctance to talk to adults about the topic, and lack of readiness from the school to support their participation. The most common forms of participation young people aspired to engage in were creating a mental health discussion group and supporting peers.

To inform the chatbot storyline, the qualitative study also examined what adolescents perceive to be the most relevant emotional difficulties and challenges to the mental health of Brazilian adolescents. Methods and results relevant to this section of the study are reported below.

A multi‐ethnic sample of 46 15–18‐year‐olds across all five regions of Brazil participated, recruited primarily through social media. Thirty‐five identified as a woman/girl (32 cisgender and three trans), six as a man/boy (all cisgender), one as non‐binary, one as fluid and three preferred not to say.

A member of the youth collaborative group conducted individual interviews and focus group discussions online, supported by a senior researcher. We combined methods for pragmatic reasons – to retain participants when only one person showed up to the focus group. We used a co‐produced interview/focus group guide that included questions such as ‘What's the mental health of Brazilian adolescents like today?’ and ‘What needs to change for Brazilian adolescents to have a good mental health?’. After each session, both facilitators agreed upon the notes taken and noted any ideas for the chat‐story inspired by the session.

Content analysis was conducted from the (detailed) session notes to identify relevant difficulties and challenges to mental health. Participants' responses were categorised and the categories identified were discussed with the team before a consensus was reached (Hill et al., 2005). The key categories and example quotes are presented in Table 2. Participants identified anxiety and depression as the most common emotional difficulties faced by Brazilian adolescents. With regards to challenges, the most frequently mentioned were prejudice and discrimination, school pressure, the COVID‐19 pandemic, unhealthy relationships, social media pressures, and pressure to conform to beauty standards.

**Table 2:** Most common categories of response from qualitative mapping of Brazilian adolescents' perceptions of common challenges to young people's mental health

Category	Example quote	Percentage of focus groups or interviews mentioning this factor
Most common difficulties		
Anxiety	“Anxiety is something that affects many young people. I think every young person is anxious nowadays. And depending on the level of anxiety, the person cannot do anything else” (FG 02)	69
Depression	“The young person isolates themselves, and the lack of communication with their parents makes them feel alone. They start keeping things to themselves, increasing the risk of developing depression” (FG 04)	38
Most common factors influencing young people's mental health		
Prejudice and discrimination (racism, LGBT + phobia, sexism)	“A person who is Black or from the LGBTQIA+ community needs twice or triple the willpower to get out of bed” (FG 03)	60
School pressure	“In school, we face a lot of pressure … to perform, to get knowledge, to achieve good grades” (FG 10)	50
COVID‐19 pandemic	“In the pandemic, many things have gotten worse … a lot of boredom; overthinking sometimes becomes a problem; worrying in advance…” (FG 13)	35
Unhealthy relationships (e.g., family members or school)	“Sometimes a person may be in an abusive relationship but because they are so immersed in it, they fail to see it and end up suffering a lot as a result” (I 1)	19
Social media pressures	“The internet is also a very toxic place. Even I'm not a big fan of digital influencers, because everything they post shows their perfect life. Someone who isn't doing well might see that and feel even worse” (FG 01)	19
Pressure to conform to beauty standards	“Issues with appearance, beauty standards – many young people who don't fit these norms end up feeling bad about themselves” (FG 12)	15

### Integrating the learnings into the chat‐story

Several design decisions were made in response to the results of our two‐staged mapping. First, given that ‘schools’ were considered to be the setting where young people felt they could make the most difference, we developed the chat‐story tool in such a way to optimise its use in school settings, while also allowing standalone use. We considered it important to seek input from teachers and other school staff to support this process; therefore, we set up a School Community Committee (see details in Part 2). To make the story more relevant to schools, we set it in a school.

In response to the common difficulties participants reported in the qualitative mapping, we decided that the main chat‐story character would experience anxiety problems, and the narrative would feature challenges frequently mentioned by participants: school pressure, prejudice and discrimination, pressure to conform to beauty standards, unhealthy relationships, and social media pressures. We left out the COVID‐19 pandemic, as the team judged that its relevance was likely to decrease over time.

Following participants' reported aspirations, we thought it was important that the chat‐story prepared players to provide peer support and to engage in collective initiatives to support their peers' mental health in school (with the creation of a mental health discussion group featured as an example). The barriers reported by participants across both studies provided the foundation for determining the chat‐story's learning goals. Given references to lack of preparedness and low self‐efficacy, the key learning goal we set was *supporting the development of skills that are relevant to peer support and collective action in mental health*.

We thought it was important for the tool to provide participants with information about mental health and sources of support for their active participation in promoting it. We included an adult figure in the story – a supportive teacher – to illustrate how to talk to adults about mental health. In response to references to the schools' lack of readiness to address young people's mental health needs, we decided to create a companion piece for teachers – a Guide for School Use – which would provide teachers with information about mental health and how to support youth participation in providing solutions to mental health problems.

## Part 2. Co‐creation: Design and refinement of the intervention

### Defining a theoretical framework

In interpreting the results of our mapping, we sought to establish a theoretical framework that would also provide a structure to define the chat‐story's learning goals more clearly. We found in Amartya Sen's Capabilities Approach (Sen, 1993) a helpful starting point. Sen argues that the realisation of one's agency depends on capabilities, including personal skills and opportunities to pursue valued goals. This means that supporting young people's participation also involves promoting the development of specific capabilities that support, motivate and enable effective participation (Pavarini, Lyreskog, Manku, Musesengwa, & Singh, 2020).

We outlined relevant skills that would enable *peer support* and *collective actions* for mental health, which were defined as the two pillars of our intervention. Peer support was defined as the process of supporting or promoting the mental health and wellbeing of another person who shares characteristics or lived experience (Mead, Hilton, & Curtis, 2001). Relevant p*eer support* skills were selected based on the literature on mental health literacy (Jorm, 2012; Nobre, Oliveira, Monteiro, Sequeira, & Ferré‐Grau, 2021; Seedaket, Turnbull, Phajan, & Wanchai, 2020), mental health first aid (Hadlaczky, Hökby, Mkrtchian, Carli, & Wasserman, 2014) and mental health peer support (King & Fazel, 2021). These included the following: identifying early signs of mental health problems, empathic listening, cultivating friendships, signposting mental health support, improving a peer's mental health awareness, asking for help, and engaging in self‐care.

Collective action was defined as the process of supporting or promoting the mental health and wellbeing of groups and communities, such as by addressing social determinants of mental health (Ionescu, Mannell, Vaughan, & Burgess, 2024; United Nations, 2020). Relevant skills for *collective action* were identified based on the literature on civic engagement (Ballard & Syme, 2016) and critical consciousness (Diemer et al., 2021; Freire, 1970, 1973), as well as UNICEF's conceptualisation of child participation (United Nations, 2020). These included identifying collective problems, knowing one's rights, teaming up with peers, forming partnerships with adults, planning actions to support the mental health of a peer collective, and balancing the pros and cons of a course of action. A more comprehensive discussion of our framework is provided by Murta et al. (2024).

### Co‐creation process

The co‐creation of the chat‐story script was an immersive process that began with an ‘Innovation Lab’ – a week‐long online programme where the three co‐design groups (researchers, youth collaborative group and creative health‐tech partners) worked together. The Lab combined improvisation and strategic, goal‐focused activities. Following the Lab, we took stock of the results of the mapping phase and reached a consensus on the following: the themes and topics to be covered in the narrative; the main learning goals; and the general structure of the chat‐story (linear with achievements). During the Lab, we brainstormed phrasal verbs, expressions, cultural concepts and memes related to mental health and youth participation; and ‘translated’ the key skills on which we were basing the learning goals into lay language to reach a common understanding amongst team members.

**Figure 2:**
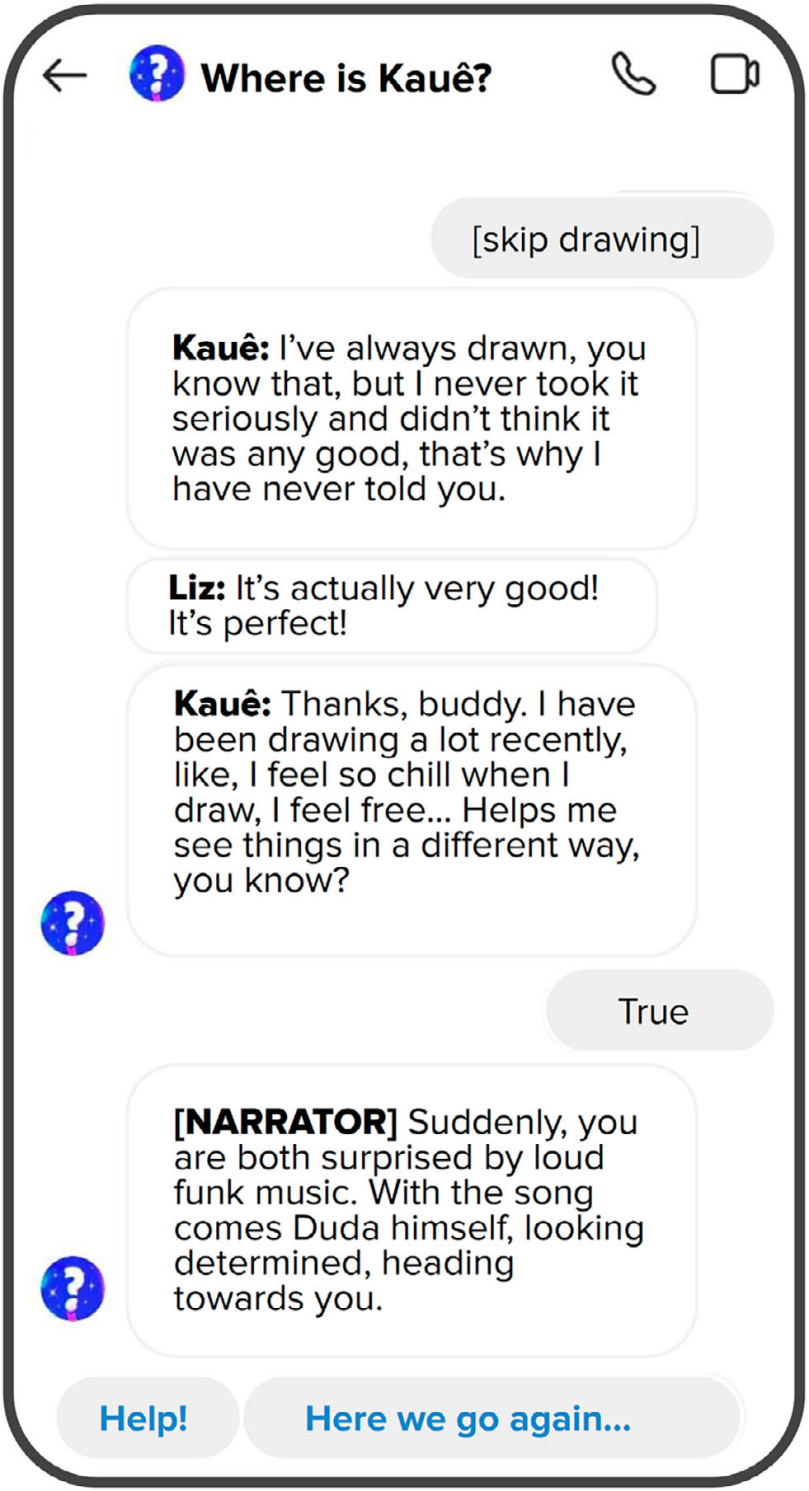
Screenshot of a translation of the chat‐story

The co‐design team continued collaborating through weekly or biweekly meetings that involved joint creation and several rounds of feedback between December 2021 and June 2022. The three groups collaborated closely, and all members provided substantial contributions across all stages of co‐creation. This collaboration progressed through four stages:

- *Setting the scene*: Team members engaged with five existing chat‐stories and individually produced outline proposals for our chat‐story. On the basis of a discussion of likes and dislikes, we converged on the key approaches for our own chat‐story: adopting language and scenarios that were realistic, naturalistic and culturally appropriate; having the story highlight the potential of schools (and teachers) to support youth participation, rather than showcasing weaknesses/barriers; avoiding heavy themes (e.g., abuse and sexual harassment); making the story light‐hearted despite the serious topics involved; having multiple, relatable characters that represented different socioeconomic and ethnic backgrounds, genders and sexual orientations; ensuring that player choices had ‘consequences’ for the story/ending; and having players choose from buttons with predefined answers, rather than type free text (see example in Figure 2), to prevent errors in communication.
- *Characters and outline*: Using the mapping findings as a foundation, we developed the chat‐story characters (personality and missions/quests). We also defined the main quest for the player: *to find their best friend, Kauê, who has disappeared at the school, in time for him to perform as the main character in a school play*. We decided that every time the player encountered a character, they would unlock a video introducing that character (see example at https://www.youtube.com/shorts/x7HPr‐XxdVo).
- *Narrative structure*: We developed a narrative outline that followed a ‘hero's journey’ structure (Campbell, 1949). This is a common story archetype whereby a person (the player) receives a call to action, experiences challenges along the way, and overcomes them to become a better person. We organised the storyline to support our learning goals, with peer support skills concentrated into the initial stages of the narrative and collective action skills in the later stages. We introduced gamification into the learning process in the form of ‘potion cards,’ which players unlock when they act in ways that reflect the proficient use of a relevant skill.
- *Script development and refinement*: Following the agreed structure/outline, we developed an initial full script. This script went through over 10 rounds of revision following input from the core team and advisory groups (Part 2c), as well as data from user‐testing (Part 3) and an acceptability study (Part 4). Revisions and amendments were discussed and agreed upon by consensus (or vote if needed) by the core co‐design team.

### Stakeholder involvement

To help make the chat‐story as accessible, enjoyable and relatable as possible, we created two advisory groups. These groups helped ensure the chat‐story integrated the views and experiences of adolescents across the country as well as the perspective of school staff.

#### The Chat‐Story Advisory Committee

The Chat‐Story Advisory Committee (CAC) consisted of 24 adolescents (from the five regions of Brazil) recruited through the project's internal networks. The CAC provided feedback across six videoconferences, which were facilitated by a researcher normally alongside a youth collaborator. The committee also responded to online polls for specific input (e.g., preferred name for main character) and tested the chat‐story prototype, providing feedback on content and technical issues. CAC members were reimbursed with gift vouchers.

CAC meetings consisted of joint readings of drafts of the chat‐story script followed by open‐ended feedback on its realism, relevance and the appropriateness of its language. The group supported script development by brainstorming examples of everyday occurrences and common dialogue/phrases from their lived experience, or that of their peers, that illustrated the themes and situations we hoped to cover. The feedback we incorporated included ideas about the places young people ‘hide’ when they struggle with their mental health (e.g., school toilets); common examples of everyday sexism (e.g., making assumptions about a girl's sexual life on the basis of her clothes; toxic masculinity and the idea that “boys don't cry”); racial microaggressions (e.g., natural hair discrimination); socioeconomic difficulties relevant to mental health (e.g., not being able to afford leisure activities; considering dropping out of school because of economic problems) and bullying (e.g., inappropriate social media posts; negative comments about one's appearance). Given the breath of their national representation, the CAC's feedback also helped us adjust the language and slang terms to remove regionalisms. For examples of contributions, see Table 3.

**Table 3:** Examples of contributions to CAC meetings, by topic

Topic	Sample contribution
‘Safe space’ at school	Participant 5: I can relate so much to him [the main character] locking himself in the restroom. I go to a technical college and it's very demanding. A friend from my course became unwell, we found her in the restroom. She was sitting on the floor, and we took her to the psychologist. It was an identical situation! (Meeting #2)
Toxic masculinity	Participant 1: Feeling bad about your own body because of pressure from other people is very common. You barely get out of the house and two hundred people start talking… Participant 2: Yes… “Sure she's given it to everyone”, “She's been around…” (Meeting #3)
Racial micro‐aggressions	Participant 6: They don't say directly that it [natural afro hair] looks bad, but it's like a hidden insult, making one feel they only look good with straight hair Participant 7: They say “Oh now it's *trendy* to do that” [wear your hair in a natural afro], when people are just being the way they are… (Meeting #2)
Socio‐economic difficulties	Participant 12: I think it's much better [for the chat‐story scene] to talk about the issue of parents forcing kids to work or study at night, rather than sanitation. There were friends of mine who had to drop out of school to work. Participant 13: At school a group is going to go out somewhere, like the movies, and you can't go because you don't have enough money. “This month we don't have money for this, this month we can't do that” [imitating a parent]. This frustrates any young person. (Meeting #4)
Bullying	Participant 3: They were bullying people for being gay, for their weight, skin colour… Some students even made fake WhatsApp profiles to send hate messages in the school group chats. (Meeting #2)
Language feedback	Participant 19: About the slang, I heard some expressions that my friends and I don't use: *“estão de tiração”* [slang to express surprise and probably displeasure] is very old; we don't use “*pilhado*” [slang for agitated or angry]. Send us the list of all the slang for us to comment on. Participant 17: Slang varies a lot from region to region, we use “*tá de caô”* [slang to express disbelief]; I use *“ainda…*?*”* [slang to express agreement] (Meeting #5)
General feedback	Participant 18: I'm loving this chat‐game; I really liked the fact that you included videos and photos of each character. It really connects the player to the game. Participant 19: It grabbed my attention. I got very curious and motivated to continue the game. (Meeting #5)

#### Feedback from adult stakeholders

The School Community Committee (SCCo) was composed of 10 professionals, including teachers, school psychologists, educationalists and educational advisers, all of whom worked in state‐funded high schools in Brasília. SCCo contributions were provided through four videoconferences, facilitated by two academic researchers and at least one youth collaborator, as well as tasks in‐between meetings. In addition to SCCo, we received input from two policy advisors and a teacher who is a social media influencer. SCCo members and advisors were paid for their contributions.

This group helped brainstorm realistic ways teachers would respond to a student experiencing mental health difficulties and ways students and teachers might collaborate to organise school initiatives. This guided the creation of a realistic and relatable teacher character (for example, in our first meeting, one participant said, “A teacher who has doubts about what to do would be interesting – learning together and in partnership with the students”). They read a full draft of the script and suggested minor amendments, largely language‐based (e.g., “I think the names [of the chat‐story characters] are not very common in our context. I'd like it to be a name that resonated with our reality and students' ‘real names’”, Meeting #2). The group also helped brainstorm school and community resources to support youth participation and mental health to be included in the chat‐story script and a Users' Toolkit provided at the end of the experience (e.g., “I think it'll be necessary to engage several fronts, not only the teacher, but also the Specialised Learning Support Team, Educational Guidance Service etc., Meeting #2).

Individually or in groups, SCCo members created lesson plans that would encourage students to take forward what they learnt after engaging with the chat‐story. Seven lesson plans were generated, which formed the basis for the development of a simple, common plan for a discussion circle with the students. This lesson plan integrated a larger chat‐story Guide for School Use (Projeto Engajadamente, 2022). The guide was created by the researchers in collaboration with a teacher advisor, with input from a representative of Distrito Federal's Education Secretary, the youth collaborative group and the SCCo.

### Description of the intervention

The final intervention was named *Cadê o Kauê?* (Where is Kauê?). When used at school, the first step consists of teachers reading the Guide for School Use (Projeto Engajadamente, 2022). The guide includes information on the theoretical bases of the project (regarding social determinants of mental health and youth participation); technical information for teachers on how to use the chat‐story in class; guidance on creating a safe space for the discussion circle to be conducted after the chat‐story; and ways through which students, teachers, parents and other staff might support well‐being and youth participation in the school. When used as a standalone online intervention, the chat‐story is accessed directly by young people online and the discussion circle is not included.

**Figure 3:**
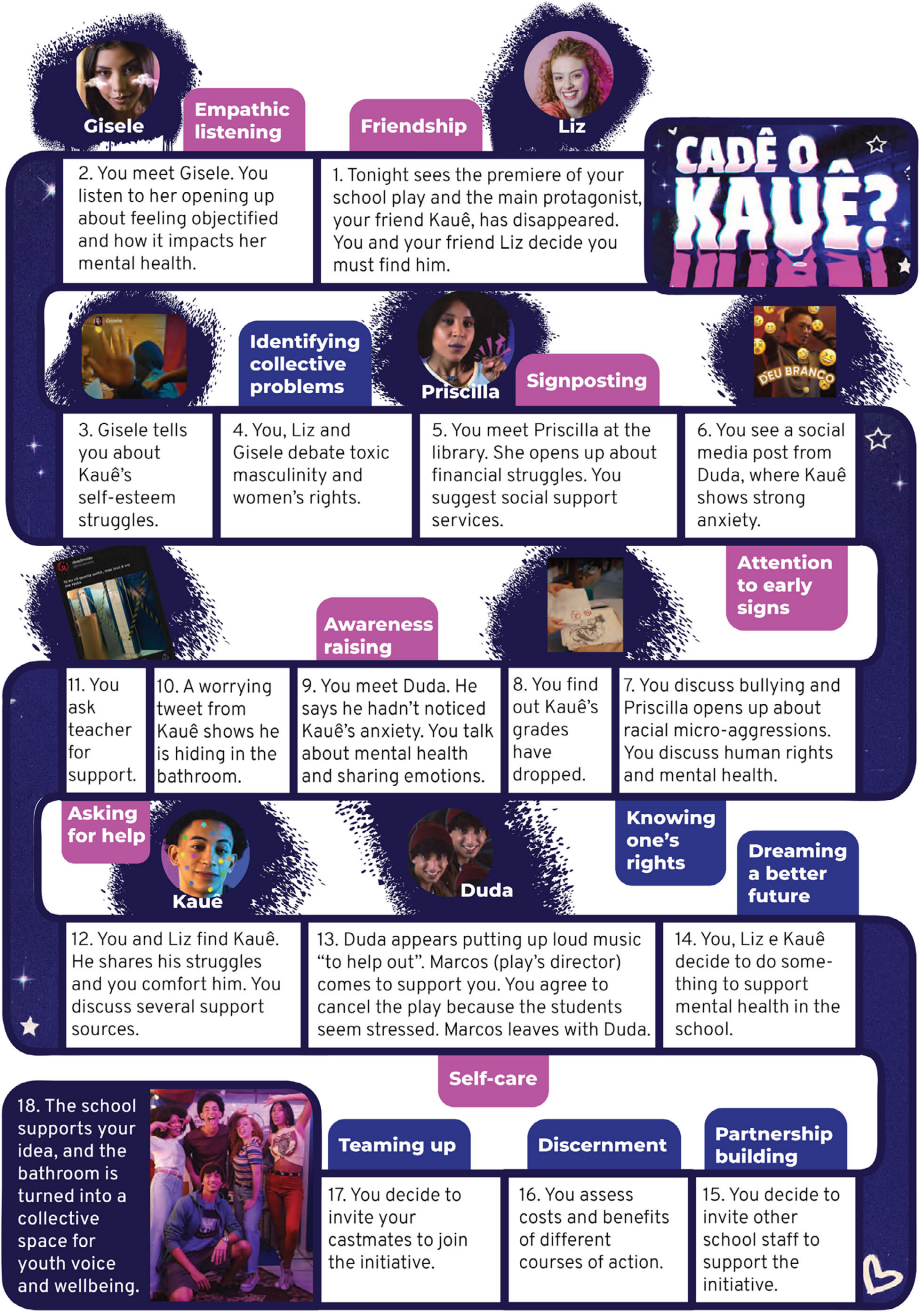
Storyline of the chat‐story, from the user's perspective, assuming they are able to unlock all skills (potion cards); the tabs refer to moments where the player is required to use peer‐support or collective action skills

Figure 3 provides a summary of the chat‐story script. The players first interact with Liz, described as their best friend, who also guides them through the story. In their joint quest to find Kauê, the player encounters other characters (peers at school) who provide clues as to why Kauê might have disappeared and his whereabouts. These clues reveal that Kauê was struggling with his mental health (e.g., there is a video of Kauê having a strong emotional reaction during a rehearsal). In conversation with each of the characters, the player learns about the difficulties these characters have experienced (e.g., racism, sexism) and support they found helpful, which raises the player's awareness of the social determinants of mental health and sources of support in the school or community. These interactions also provide opportunities for players to practice their participation skills. Eventually, the user finds Kauê hidden in the old school toilets, where they are able to offer him support. In “conversation” with Kauê and Liz, the player is led to reflect that many students in the school experience challenges, and together they decide to create a safe space in school where students can talk about things that matter to them (see closing video at https://www.youtube.com/watch?v=wTeOJCaSYAQ).

**Figure 4:**
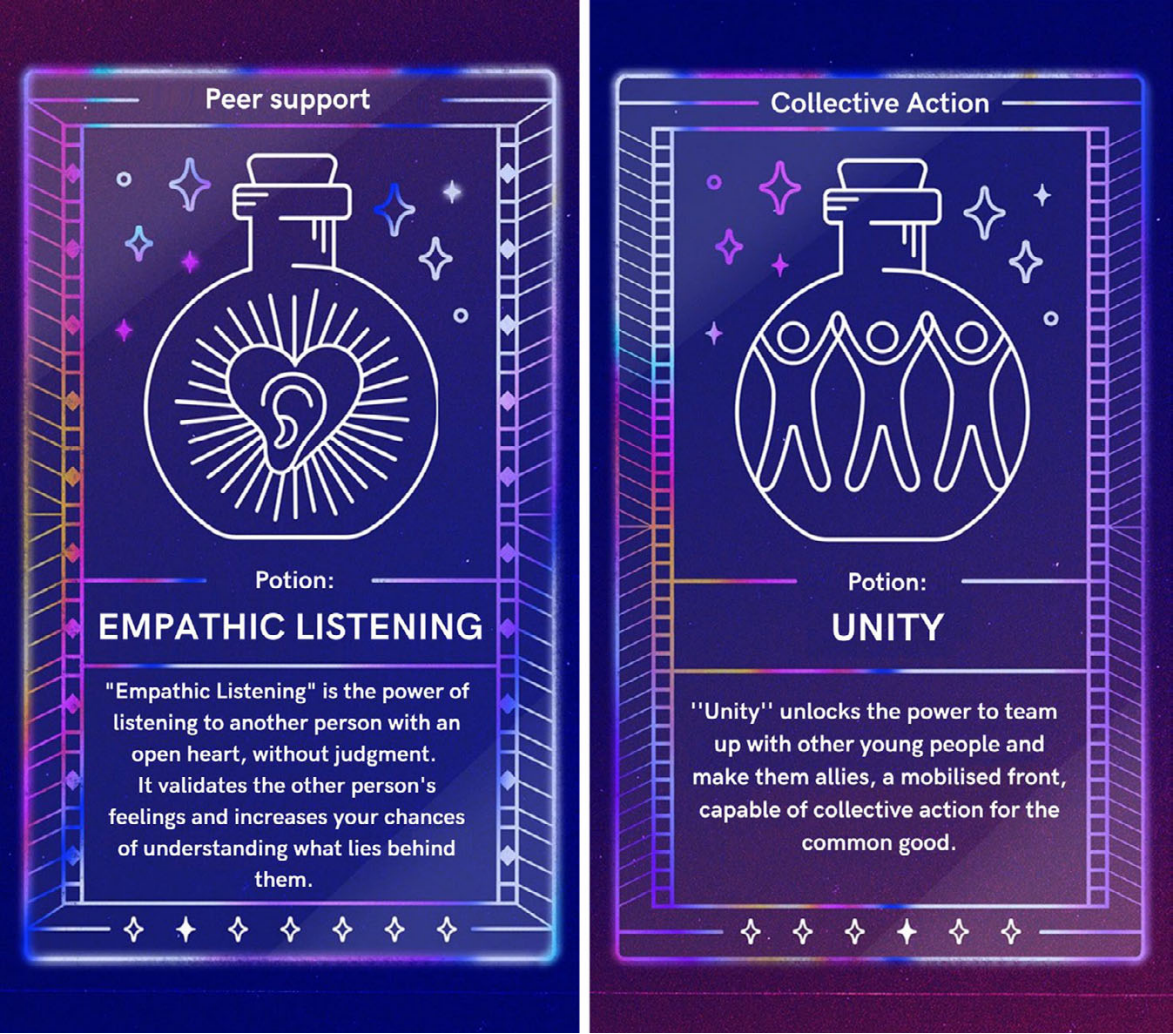
Examples of the skill (‘potion’) cards used in the chat‐story; on the left is a peer‐support card; on the right a collective action card

During the story, players unlock ‘potion cards’ when they demonstrate the proficient use of a skill. ‘Potions’ are categorised by the type of skill: peer support or collective action. Each card provides a youth‐friendly description of the skill unlocked (see Figure 4). If the player fails to unlock a card, the narrator or another character provides a model of how to use the relevant skill or talks about its importance. For instance, in one scene the player has the option to listen to a friend's problems or ‘skip’ to the next scene; those who choose to listen unlock the ‘Empathic Listening’ card. Those who skip are later presented with a video of the narrator reflecting on the fact that they chose not to listen and talking about the importance of listening (see video at https://www.youtube.com/shorts/AIko_oJxPMs).

At the end of the experience, the player receives one of four profiles, depending on the number of peer support (PS) and collective action (CA) potions they unlock: *activist* (high CA, low PS), *buddy* (high PS, low CA), *protagonist* (both high), or *chilled out* (both low), all containing an encouraging description about their potential. A toolkit in pdf (Appendix S1) is then provided with a range of community and school resources they can use to support their peers' mental health in real life (e.g., helplines, information on how to create a school council).

When the chat‐story is administered in class, the intervention includes a discussion circle during a lesson subsequent to that where they used the chat‐story. The discussion is guided by the following three questions: “What can we do to ensure no student feels the need to hide in the bathroom, like Kauê did? – how can we support one another?”, “Is there something we can do to support mental health in our classroom and school?”, “Whom and what can we count on to implement these actions?”

### Audio‐visual production and technical development

When the final version of the script had been agreed upon, the chat‐story was integrated into a chatbot format and the accompanying audio‐visual material was produced. This included videos, images, and posters. All audio‐visual production was led by Talk2U and partners, based on the script, text and layout co‐produced by them, the researchers and the youth collaborators. The youth collaborative group and researchers also provided visual source material (memes, videos, or images) for inspiration, as well as input and feedback on draft versions. At this stage, the fully designed and typeset potion cards, the User's Toolkit (Appendix S1) and the Guide for School Use (Appendix S2) were also produced. These materials were scripted by the researchers with input from the core team and advisors. The chat‐story was then integrated into Telegram and Facebook Messenger.

## Part 3: User testing

### User testing session 1

#### Methods

Thirty students (15 girls/women, 12 boys/men, 1 non‐binary, 1 prefer not to say) aged 16–18 years in one state school in Brasilia participated in a user‐testing session. The chat‐story was independently administered by the teacher in class. Feedback was gathered through a survey included at the end of the experience and an interview with the teacher.

#### Key learnings

The chat‐story was too long to be administered in a double lesson (1h30m); only half of the students managed to complete it (also, for that reason, only 15 managed to fill out the feedback survey). The teacher noted that most students did not have Telegram or Messenger accounts and were unwilling to download a new application because of limited storage space on their phones. The student feedback was positive, notably with regards to usability (*M* = 8.67/10, *SD* = 1.45) and enjoyment of the role‐play (*M* = 8.87/10, *SD* = 1.77). Seven out of 15 participants thought the chat‐story touched on sensitive topics. Additional survey results are provided in Appendix S3.

In response to these results, we integrated the bot as a widget on our website at www.engajadamente.org. Feedback from SCCo also supported this, as they reported that many schools had firewalls blocking some aspects of social media. By the time we had finished this pilot, Instagram had launched its chatbot integration feature, so we also integrated the chat‐story onto Instagram's Messaging (@kade_o_kaue). We also reduced the length of the script/experience.

Given the important changes incorporated after this session, the feedback on ‘sensitive topics’ and the high attrition (which potentially biased the feedback received), we conducted a second user‐testing session.

### User testing session 2

#### Methods

Seventeen students enrolled in the last year of compulsory education (7 boys/men and 10 girls/women) in a state school in Brasília completed the chat‐story via the website or Instagram, administered by the classroom teacher. After the chat‐story a semi‐structured group discussion was co‐led by a researcher and a member of the youth collaborative group, covering general experience, accessibility, language and any potential emotional triggers. The academic researchers and youth collaborators jointly analysed the session notes, with a view to identifying key insights and actionable feedback.

#### Key learnings

Participating students managed to access the chat‐story and complete it via the website or Instagram. All students provided positive feedback (e.g., “it was cool”, “I liked the unity [among characters]”, “it gave me a sense of hope”, “the tool helps us to help”); the language was described as “accessible”, “easy to understand” and “current”. Nine out of 17 participants reported a bug on the final messages sent by the bot, which we fixed following the session.

Fifteen students identified no emotional triggers, but two referred to a scene where the user has the option to ask Kauê whether he self‐harmed as potentially evocative. In the discussion, the students agreed that the scene was acceptable and it was important to include it. This feedback was consistent with that of the youth collaborative team and the Chat‐Story Advisory Committee (who originally proposed it). Given this input and evidence suggesting that these questions should not be avoided just because they are difficult (Dazzi et al., 2014), we decided to keep that scene as designed. We introduced a feature that allowed users to ask for support at any point by typing ‘Me ajuda’ (‘Help me’). This unlocks a supportive message, including mental health helplines.

Following the user‐testing sessions, we progressed to an acceptability study to gather qualitative feedback on the chat‐story from a larger number of participants, and also evaluate the discussion circle that accompanies the chat‐story when administered in schools.

## Part 4. Acceptability testing

### Methodology

We conducted a study to gather qualitative data on the acceptability to young people of both the chat‐story and the discussion circle in schools. The study was embedded in a larger study testing the feasibility of a randomised trial, the results of which are reported elsewhere (Pavarini, Murta, & Mendes, 2024). State high schools in Brasília were invited to participate via a phone call, email or in‐person visit. Participating teachers (1 or 2 per school) attended an in‐person meeting where all materials were distributed (i.e., information sheets, consent forms, earphones and internet routers if needed) and instructions were given on how to run the intervention and administer questionnaires. In this qualitative study, we included the 11 schools that were randomly assigned to participate in the ‘Cadê o Kauê?’ intervention and answer an open‐ended survey, from a total of 22 schools that participated in the larger study. Although the intervention was made available to the control group schools at the end of the study, their involvement was optional, and no feedback was gathered.

Teachers ran the chat‐story in class (students who were not able to complete it in class were provided with a link to complete it at home); in a subsequent class, teachers facilitated the conversation circle in accordance with the instructions provided in the Manual for School Use. A questionnaire was administered in class up to 2 weeks after the conversation circle, which included open‐ended questions for participants to indicate what they ‘liked’ and what they ‘would change’ about the chat‐story and the conversation circle. No reimbursement was provided.

### Data analysis

Data were analysed using content analysis. Two researchers independently generated initial categories based on the full dataset; these categories were then compared and refined in discussion with the team. The final coding scheme was applied by one researcher and independently checked by at least one other team member, with disagreements resolved by consensus.

### Results

Seven schools delivered the intervention and administered the post‐intervention questionnaire (the other four schools dropped out at different points due to time constraints or changes in schedule), totalling 138 students. Participants were aged 16.2 years on average (*SD* = 1.00); in terms of gender, 60.1% identified as a girl/woman, 33.3% as a boy/man, 1.4% were non‐binary, 0.7% chose ‘other’ gender and 4.3% did not report.

Eighty‐seven percent (120/138 students) took part in the chat‐story, out of whom 76.7% (*n* = 92) reached the final chapter, as reported by the student. Table 4 presents the response categories for ‘likes’ and suggested changes, alongside quotes and frequencies. Participants appreciated the following aspects of the chat‐story: (1) certain aspects of the user experience (e.g., the interactivity, the ‘potions’ and the user‐friendliness); (2) the authenticity of the narrative, characters and topics covered; and/or (3) the chance to learn about mental health or the actual learning derived from the experience. Others provided unspecific positive answers. About half of the participants said there was nothing they would change about the chat‐story; the second‐most‐common feedback was the chat‐story's length (i.e., the experience or specific interactions taking too long).

**Table 4:** Chat‐story and conversation circle: Categories coded from participants' responses about features they liked and changes they would propose to the chat‐story. Answers could fall under more than one category

Chat‐story			
Type of response and category		Sample quotation	Frequency (%)
Likes (*n* = 120)	User experience elements	“It's very similar to an RPG [role‐playing game], the ‘potions’ we receive depend on what we do in the story”	33.3
	Authenticity of topics, narrative, characters	“The story addresses real problems, lived daily by many of us”	28.3
	Unspecific positive feedback	“Very cool”	24.2
	Learnings	“I realised that helping those around us is the best thing we can do”	12.5
	No/Nothing	“Not much”	3.3
	Missing answers	N/A	2.5
Proposed changes (*n* = 120)	Nothing	“Nothing 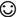 ”	49.2
	Length	“I'd change the length of the activity, because it became a bit tiring”	12.5
	Response options	“I'd add more response options, with different types of humour”	11.7
	Connectivity issues	“The speed, because when you answered there was a delay, but it was a great experience”	8.3
	Technical suggestions	“To be able to answer through other apps like whatsapp”	5.8
	Other	“Some things”	5.0
	Narrative	“Kauê's story”	4.2
	Unsure	“honestly don't know”	3.3
	Missing answers	N/A	1.7

Conversation circle			
Category		Example quote	Frequency (%)
Likes (*n* = 108)	Supportive interactions	“That everyone opened up to the conversation showing their feelings just like Kaue”	38
	Unspecific positive feedback	“I enjoyed everything, it was very interesting”	25
	Learnings	“Interesting because it clarified a lot of things about mental health”	18.5
	Topic	“It covered really important topics, which shouldn't be neglected”	9.3
	Negative aspects	“I didn't like it very much”	6.5
	Missing answers	N/A	3.7
	Other	“More or less”	1.9
Proposed changes (*n* = 108)	Nothing	“Nothing, I liked the way it went”	63.9
	Student participation	“More student participation, less shyness etc” (sic)	21.3
	Unsure	“Don't know”	4.6
	Other	Not doing it during chemistry class, because I had an exam on that subject on the day!!!	3.7
	Facilitator	“The educator who participated in the conversation circle – no empathy and unprepared for these conversations”	2.8
	Setting	“The place where it was. It could be a more comfortable place so we could talk better”	1.9
	Missing answers	N/A	1.9

A total of 108 out of 138 participants (78.3%) took part in the conversation circle. Table 4 shows all response categories, quotes and frequencies. Main ‘likes’ included (1) the open, accepting and non‐judgmental environment in which to share one's experiences and feelings or listen to those of others; (2) lessons derived from the chat‐story such as information about mental health and strategies to engage in peer support and collective action; and (3) the theme of mental health and the topics raised during the conversation. There were also unspecific positive responses. Over two‐fifths of participants indicated there was nothing they wanted to change about the circle; the second most common response was to increase the participation, openness or attitude of the students taking part.

### Key learnings

Following these results, we further reduced the chat‐story's length. Because the technical issues reported at this stage were related to Internet connectivity, we did not make further changes to the tool. Considering the positive results from this study, we decided to progress to an online dissemination of the chat‐story along an embedded survey (Part 5), while planning a further evaluation within the school environment.

## Part 5. Dissemination

A social media campaign was implemented, with online ads developed by Talk2U with input from the researchers and youth collaborative group, as well as a social media influencer post. These posts and advertisements targeted adolescents aged 15–18 years across all Brazilian regions. During the campaign, the chat‐story was freely available online and accessible via Instagram, Facebook Messenger and the project's website widget. The advertisements directed users to the chat platform, where they could access *Cadê o Kauê?* after consenting to the terms and conditions and confirming they were 15 years or older. At the start of the experience, users were also asked to provide short demographics.

A short optional survey was embedded at the end of the experience to obtain initial data on the chat‐story's efficacy as well as further user feedback. The survey consisted of five questions, two of which measured self‐efficacy: “Do you feel *more* or *less* prepared to: (1) ‘help friends who are going through mental health difficulties?’ and (2) ‘organise or take part in action to promote mental health in your school’” (for each item, students had to choose a rating from 1 to 5, with 1 being ‘less prepared’ and 5 ‘more prepared’). Two other questions measured whether they felt *more* or *less* motivated to (1) ‘help friends who are going through mental health difficulties?’ and (2) ‘organise or take part in action to promote mental health in your school’ (again the ratings ranged from 1, *much less motivated*, to 5, *much more motivated*). One question measured attitudes/openness in relation to mental health after playing the game (with ratings ranging from 1 *much less open* to 5 *much more open*) and one question measured the acceptability of using a chat‐story to talk about mental health (with ratings ranging from 1, *terrible idea*, to 5 *great idea*). An open question at the end encouraged users to openly share any ‘opinion or suggestion’ they had on the chat‐story. No reimbursement was provided to survey respondents.

### Results

The final chat‐story was accessed by 5,402 players. Out of this total, we excluded participants who either: indicated being out of Brazil (*n* = 25); did not report their country of residence (*n* = 832); were older than 18 (*n* = 746); or did not report their specific age (*n* = 211). This resulted in a final sample of 3,949 adolescents in Brazil between 15 and 18 years of age. Out of this sample, 795 participants (*M*_age_ = 16.7 years, *SD* = 1.34) from all five Brazilian macro‐regions completed the survey (64.4% girls/women; 31.2% of boys/men; and 4.4% non‐binary).

Participants, on average, chose the option indicating that the chat‐story increased their motivation to support their peers (*M* = 4.54, *SD* = 0.80) and to engage in collective action for mental health (*M* = 4.27, *SD* = 0.82). Participants also selected the option indicating that the chat‐story made them feel better prepared to help their peers (*M* = 4.49, *SD* = 0.82) and to engage in collective action for mental health (*M* = 4.20, *SD* = 0.97). Finally, participants, on average, selected the option indicating that the chat‐story increased their openness about mental health (*M* = 4.29, *SD* = 0.83) and that the chat‐story was ‘a great idea’ (*M* = 4.56, *SD* = 0.86), suggesting high acceptability.

Out of those who completed the survey, 411 players left opinions or suggestions in the open field, which were coded using content analysis. A researcher developed an initial coding scheme, which was iterated and refined in discussion with the team. The final scheme was applied to all responses and checked by two independent researchers, with disagreements resolved by consensus. As shown in Table 5, the response categories included the following: (1) unspecific expressions of approval, commendation or admiration; (2) suggestions for expansion (e.g., additional chapters; new chat‐stories covering other topics; versions for younger adolescents or adults); (3) references to lessons or benefits the chat‐story brings; (4) references to authenticity, feeling immersed in the chat‐story environment and the player's identification with the narrative and its characters; (5) praise for the chat‐story as a method and user‐experience elements such as interactivity, ‘potions’ and audio‐visual pieces; (6) suggestions for improving the narrative or technical features; (7) the relevance of the topic covered (mental health); (8) implementation considerations such as suggestions to disseminate the chat‐story to schools, private companies, etc.; (9) other miscellaneous entries; (10) feedback on technical glitches; (11) negative aspects of the experience.

**Table 5:** Main categories of responses received during dissemination campaign, with sample quotations and frequency

Category	Sample quotation	Frequency (%)
Approval, commendations and admiration	“That was so cool!! Congratulations on the amazing project 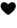 ”	34.8
2 Expansions	“Talking about world problems like deforestation, pollution, racism, poverty, hunger like the UN list with its 17 goals I think would make a great chat story and your work was very well done. Thank you for everything. I was feeling kind of bad but after that [playing] I feel a little better”	18.5
3 Learnings and benefits	“Well, this chat helped me a lot to understand that I need to be more open about what I feel and talk more with my friends about my feelings and especially about my mental health. Oh! And also, to be much more attentive to other people's feelings and mental health as well. Now I know I can better understand what I feel and what others feel. I've always wanted to be someone people can lean on, and now, I'm even more motivated to be that someone 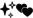 !!!”	15.6
4 Authenticity, immersion and identification	“I thought the project was wonderful! Very well done, recorded, narrated, scripted and edited! I really liked the characters and how they approached their respective problems, some of them REAL but not talked about (like Priscilla's situation). Thanks for the experience. 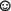 ”	10.9
5 Chat‐story method and user‐experience	“I thought your project was really cool! It's great to talk about these subjects, even more so in a fun way like this. I really liked it. The ‘potions’ are something very cool and the videos when you don't reach the necessary points are also very cool! Congratulations there for you. Liked a lot:)”	10.7
6 Suggestions for improvement	“I LOOVED IT!!! Super creative and fun. The plot‐twist at the end with Liz [revealing she is the narrator] made my jaw drop! Hahaha. I suggest using more plot‐twists. This makes the experience much more exciting Xd. Thank you so much for the experience 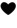 ”	11.9
7 Theme relevance	“I found it a very interesting and creative initiative. It is always important to talk about mental health, especially in the school environment, where young people spend most of their time”	10.5
8 Implementation considerations	“Such a beautiful idea this story. All schools could have this and that would be awesome for all students”	4.9
9 Other	“Hello”	2.7
10 Technical glitches	“The idea and the way it is done are great, but you should remember that from time to time Insta has its bugs, as happened to mine a few times during the story, so always leave warnings that these errors can happen, because sometimes the message wouldn't come (sometimes it only showed when I sent something) and the person may end up giving up the interaction for not knowing it”	2.4
11 Negative aspects	“This looks like government propaganda. Improve the scripts, pls”	1.5

The response categories were derived from players’ open‐ended feedback using content analysis. Percentages are out of the total number of players who left opinions or suggestions in the open field (*n* = 411). Answers could fall under more than one category.

## Discussion

This paper has described the iterative development, acceptability testing, and online dissemination of *Cadê o Kauê?*, a chat‐story intervention to enhance young people's participation in promoting mental health and well‐being within their peer communities in Brazil. The tool resulted from a sustained, in‐depth co‐design process amongst academic researchers, a youth collaborative group and health‐tech/creative industry experts. The co‐design was guided by a mapping phase suggesting that young people aspired to support their peers and set up mental health initiatives in schools, but lacked the skill and confidence to do so. Several rounds of input from stakeholder groups, including not only youth advisors and user testers, but also young people's advocates (e.g., teachers, adolescent health policy experts), enabled the development of a relatable and accessible tool. The resulting narrative induces players to investigate the disappearance of their friend Kauê and to find out where he is and what's happened to him, before the school play starts, where he is the main character. Via interactions with characters, the player learns that their friend is struggling with his mental health and ‘unlocks’ skills (e.g., signposting, establishing partnerships) that enable them to support him and others. The chat‐story received positive feedback from students, who praised its authenticity and suitability. They also showed appreciation for the learnings derived from the experience. The final chat‐story was disseminated online along an embedded survey. The results of the survey indicated that adolescent users perceived the tool as increasing their motivation and self‐efficacy for peer support and collective action for mental health, as well as enhancing their openness about mental health.

A notable feature of *Cadê o Kauê?* is its focus on promoting youth participation in supporting their peers' mental health, rather than directly promoting players' own mental health and wellbeing. This represents a highly scalable intervention model, which leverages and strengthens peer‐to‐peer relationships and community ties as well as young people's activism and social action. In this way, the intervention provides a creative and engaging alternative to peer support and youth empowerment training, which have so far yielded inconsistent results (see King & Fazel, 2021; Morton & Montgomery, 2013). The element of capacity‐building for collective action in mental health promotion is pioneering, and equally distinctive is our intervention's focus on promoting democratic values and raising awareness of the social determinants of mental health, rather than taking an approach focused on the individual. From that standpoint, *Cadê o Kauê?* joins the growing field of socially oriented games (Flanagan, 2018; Flanagan & Nissenbaum, 2014), which incorporate activist themes and promote communal values.

Most digital mental health tools are standalone, direct‐to‐consumer applications available online (Anthes, 2016). However, there have been calls, not only for better integration but also for increased relevance of these tools within larger systems of care (Roland, Lawrance, Insel, & Christensen, 2020). Even though our chat‐story was originally envisioned for standalone use only, input from young people suggested that *schools* were where they could make the most difference. This led our team to shift their focus and make the chat‐story relevant and applicable to state high schools. We centred the narrative around school experiences and co‐created a companion piece for teachers to support classroom use, in addition to direct online use. We also engaged policy consultants to ensure alignment with Brazilian policies (Lordello, Brambatti, Murta, Siston, Mendes, & Pavarini, 2023). This process illustrates the importance of active stakeholder involvement as well as flexibility and adaptability on the part of the creators to ensure the technology aligns with community priorities and the context in which it is embedded, as highlighted by several researchers (Hawkins et al., 2017; Zidaru, Morrow, & Stockley, 2021).

In this project, our top priority was to provide a tool to help address the urgent mental health needs of young Brazilians post‐pandemic while promoting citizenship. Consequently, we chose to do an online release of Cadê o Kauê? without waiting for a thorough efficacy evaluation, as obtaining funding for and conducting a trial in Brazil would have taken a substantial amount of time. Our immersive co‐design process, along with highly positive results from the acceptability study, instilled confidence that the tool would be well‐received by Brazilian adolescents without causing harm. With encouraging results from the campaign, plans are underway for a school trial and subsequent implementation. The tension between the need for quick dissemination of digital interventions to meet local needs and robust prior evidence is a common challenge in digital mental health, as observed by Pavarini, Reay, et al. (2024).

### Limitations

Even though an effort was made to represent a wide range of young people in user involvement and chat‐story characters, Brazil is a highly heterogenous country and it was not possible to cover all experiences, backgrounds and contexts. For instance, the specific challenges of Brazilian adolescents from indigenous backgrounds were not addressed, and the role of parents/guardians is only briefly touched upon.

Initial evidence of efficacy in Part 5 was restricted to players who completed the full chat‐story online and chose to answer the optional survey. This likely biased the results towards participants who enjoyed the experience. The response rate was low (20%) and based on a single‐point, single‐group response (rather than pre‐post and/or with a comparator), which would have provided a more rigorous examination of efficacy. The format of delivery also differed from that intended for school use, which also includes a teacher‐led discussion component. Future research will test the intervention's impact on young people's participation skills and mental health using more robust designs and across settings. It is also worth noting that, in its current form, chat‐story implementation might be restricted in contexts with limited Internet or device access. In our evaluation studies, for example, mitigation measures had to be adopted (providing a router).

Finally, because youth participation is also dependent on conducive environments, such as supportive policies and partnership from adult stakeholders (United Nations, 2020; Vijayaraghavan et al., 2022), it will be important to ascertain the efficacy of the chat‐story in the context of potential socio‐structural barriers.

### Learnings from co‐design

*Cadê o Kauê?* was made possible by the openness, flexibility and trust of multiple stakeholders who brought their unique experiences, expertise and skills to an immersive co‐design process. Our ‘radical co‐production’ approach prioritised young people's views and needs by not just ‘consulting’ them but by fostering deep involvement. Co‐production spanned from priority setting to dissemination, incorporating real‐life stories and engaging school teachers and policy actors. Joint leadership amongst academic researchers, young people, and health‐tech experts, supported by a network of advisors, presented several challenges. We navigated differences in expertise, experience, opinion, priorities, institutional power, and familiarity with the co‐production model, amongst others. Our key takeaway is that immersive co‐design requires significant time and resources to be done thoughtfully, including ongoing training, reflection, and adjustment (Siston et al., 2023). It demands not only practical solutions like voting and structured feedback, but also virtues like humility, transparency, trust, and care, which we have cultivated through the process.

## Conclusion

Stories inspire, engage, and compel us to think or behave differently. In *Cadê o Kauê?*, a gamified chat‐story helps build young people's capacity to support the mental health of their friends and their school collective, helping address the significant mental health challenges prevalent in Brazil (Zuccolo et al., 2022). Against a backdrop of political and social challenges, including compromised children's rights and escalating school violence (Ferreira, Dos Santos, & Oriente, 2023; Oliveira, 2022), this creative intervention assumes critical importance. Through both its aims and the process of co‐creation, the intervention aims to foster collaborative cultures and promote adolescents' citizenship. Co‐design for delivery at pace and scale is not without challenges, especially in LMICs with infrastructure constraints and unstable socio‐political environments. Yet, close collaboration amongst researchers, young people and creative health‐tech partners can be transformative. The powerful convergence of diverse disciplines, expertise, and creativity holds the potential to generate interventions that are attractive and novel, while also being theoretically robust and sustainable, supporting real‐world application and wide deployment.

## Ethical considerations

The project received ethics approval from the Oxford Tropical Research Ethics Committee (Ref. 501‐21, Ref. 550‐21), the University of Brasilia Humanities and Social Sciences Ethics Committee (Ref. 4.688.652, Ref. 5.196.133) and the Brazilian National Ethics Commission (Ref. 4.932.068, Ref. 5.510.769). Informed consent was gained from all participants or their carers (with young people's assent) for each stage of the project.

**Key points**

- The authors co‐designed a digital capacity‐building intervention in the form of a “chat‐story”, aimed at enhancing youth participation in the promotion of mental health in Brazil. The chat‐story aligns with the priorities of adolescents, fostering skills for peer support and collective action in school environments.
- The approach involved a comprehensive co‐production effort, bringing together researchers, youth collaborators, and creative/health‐tech experts. The authors also actively engaged with various advisory groups, including adolescents and their advocates, such as teachers and policy experts.
- The resulting chat‐story resonated with adolescents, who found it engaging and authentic, as well as effective in strengthening their motivation to promote their peers' mental health and their ability to do so. As such, this work serves as a model for co‐created digital mental health interventions that prioritise youth agency, community bonds, and storytelling.

## Supporting information


**Appendix S1.** User's toolkit.

**Appendix S2.** Guide for school use.

**Appendix S3.** User testing session 1 additional survey results.

## Data Availability

The data that support the findings of this study are available from the corresponding author upon reasonable request.
