# Supplementary material for: Cadê o Kauê? Co‐design and acceptability testing of a chat‐story aimed at enhancing youth participation in the promotion of mental health in Brazil
Source: J Child Psychol Psychiatry. 2024 Dec 20;66(5):697–715. doi: 10.1111/jcpp.14078 (PMC12018289; doi:10.1111/jcpp.14078)
Supplement: Supplementary file 1 — Appendix S1. User's toolkit. [file JCPP-66-697-s003.pdf]

TÁ, MAS E ONDE BUSCAR AJUDA?
CONHEÇA ALGUMAS
FERRAMENTAS DE APOIO

CADÊ O KAUÊ?

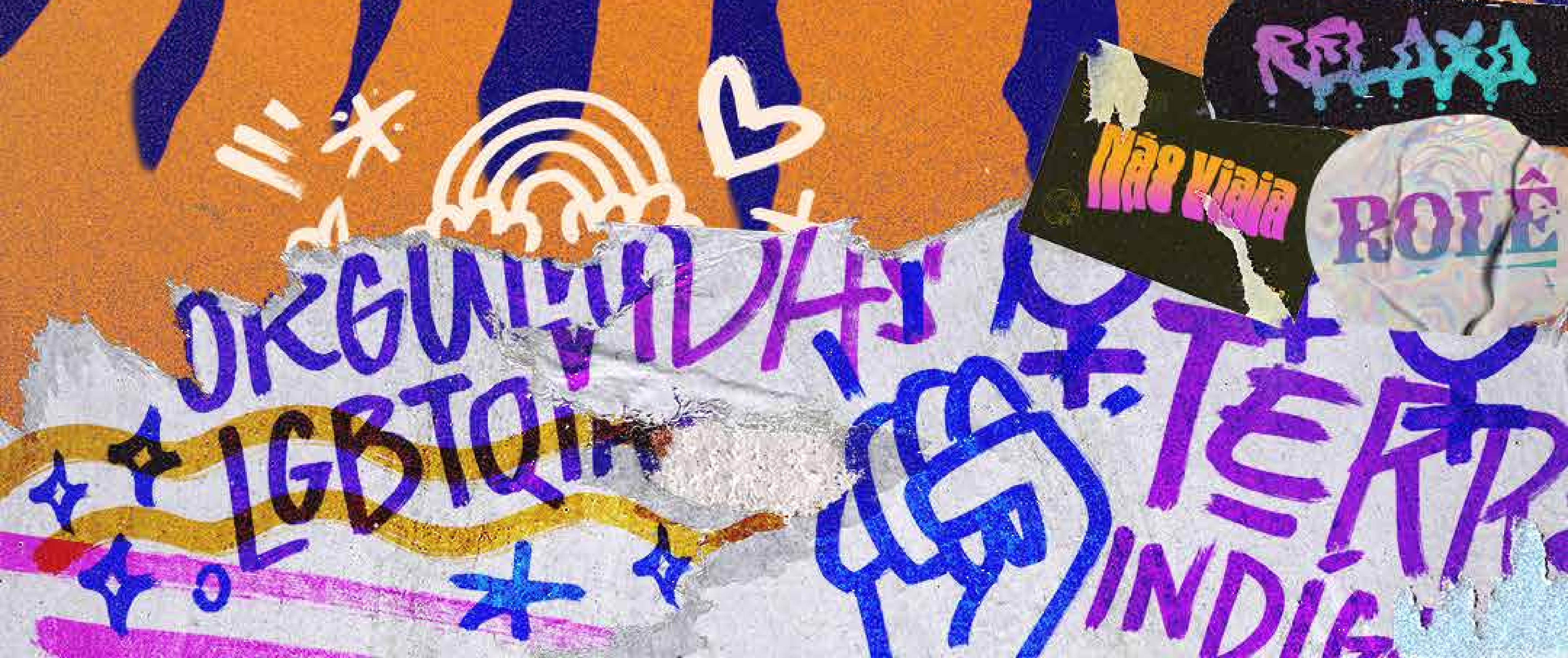

APOIO AOS AMIGOS

Às vezes, a gente quer ajudar alguém ou até a gente mesmo, mas não sabe bem o que fazer, né?

A lista de serviços abaixo apresenta boas opções para buscar ajuda para questões de saúde mental, bem-estar e violação de direitos:

- **Centro de Valorização da Vida (CVV), Disque 188:** é uma opção de ajuda imediata. Você ou quem estiver precisando pode ligar nesse número e falar com alguém que estará disposto a te ouvir a te ajudar a passar por aquele momento difícil. O CVV também possui um serviço de chat: www.cvv.org.br/chat

- **CAPsi:** O Centro de Atenção Psicossocial (CAPs) oferece serviços gratuitos de caráter aberto e comunitário. O CAPsi é especializado em adolescentes e jovens. Descubra o mais próximo de você com a ajuda do número 136.

• **Mapa da Saúde Mental:**
Você também pode acessar o site www.mapasaudemental.com.br e ver qual o serviço de saúde mental mais próximo de você. O serviço é gratuito!

• **Pode Falar!:** este é um canal de ajuda em saúde mental para você que tem de 13 a 24 anos. Para mais informações, acesse: www.podefalar.org.br

• **Clínicas-escola de Psicologia:**
todas as faculdades de Psicologia têm uma “clínica-escola” que oferece atendimento psicológico gratuito ou a preço social.
Os atendimentos são feitos por estudantes de Psicologia no último ano do curso e sob a supervisão de professores experientes.
Se você precisar de atendimento psicológico, ou conhece alguém que precise, mas não tem condições de pagar, essa pode ser uma boa opção. Procure a faculdade de psicologia mais perto de você e se informe sobre esse serviço.

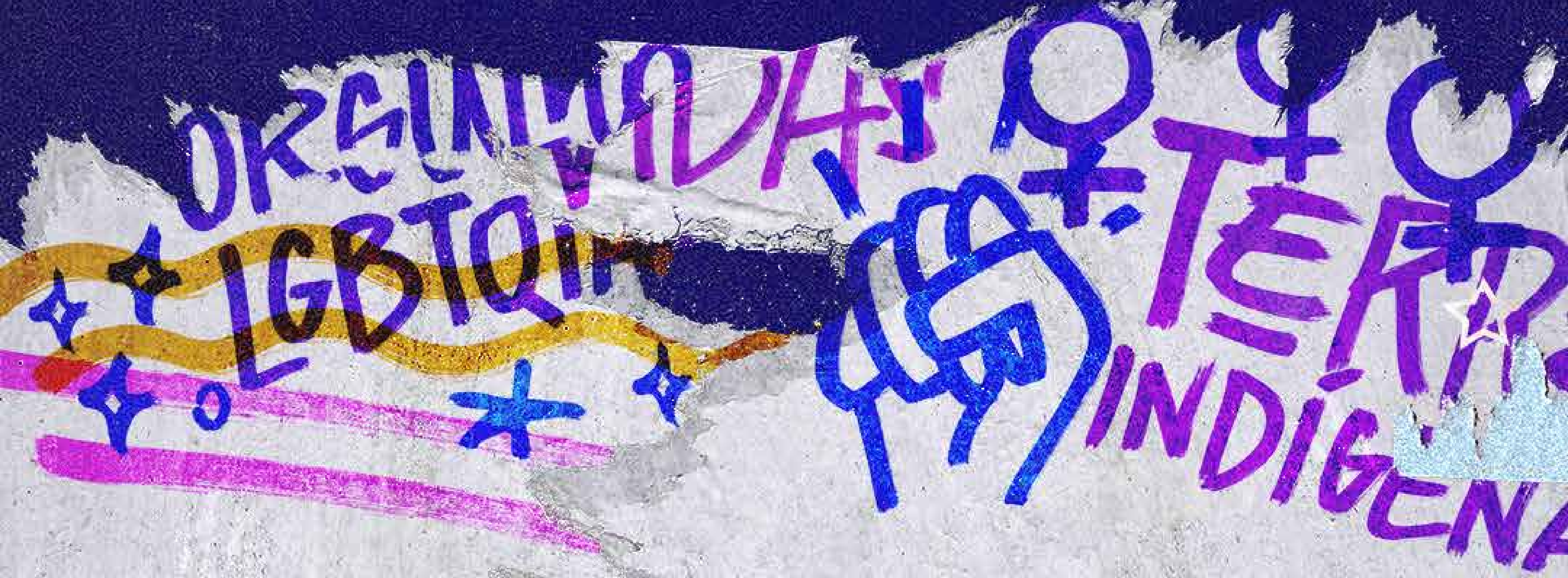

• **Geração Zelo:** a ASEC - Movimento Saber Lidar, em parceria com o UNICEF, oferece trilhas gratuitas e online com desafios criados de jovem para jovem, onde você poderá aprender como se cuidar, cuidar das suas relações e lidar com dificuldades. Para mais informações, acesse: www.asecbrasil-ead.org.br/mod/page/view.php?id=6

• **Conselho Tutelar:** o Conselho Tutelar existe para proteger os direitos das crianças e dos adolescentes. Caso você conheça alguma criança/adolescente que esteja sendo mal tratada e/ou esteja passando por alguma necessidade, você pode procurar o Conselho Tutelar da sua cidade. Para obter mais informações do Conselho Tutelar da sua cidade, ligue no 156.

• **Disque 100:** as denúncias feitas neste número são encaminhadas para os órgãos de proteção. Podem ser feitas denúncias de violações de direitos de crianças e adolescentes, pessoas idosas, pessoas com deficiência, população LGBT, população em situação de rua, entre outros. Você também pode denunciar pelo app, basta buscar no Google 'Direitoshumanosbrasilbot' e depois baixar e instalar o app.

• **Ligue 180:** este número é a 'Central de Atendimento à Mulher'. Nesta central, as mulheres em situação de violência recebem escuta e acolhida qualificadas. Este serviço registra e encaminha denúncias de violência contra a mulher aos órgãos competentes.

• **SaferNet:** nesta plataforma, você poderá denunciar casos de bullying online. Você também encontrará informações para quem tem dúvidas sobre as violências cometidas pela internet. Para mais detalhes, acesse: https://new.safernet.org.br/

APOIO COLETIVO

Saber onde buscar ajuda é extremamente importante, né? Mas como podemos fortalecer as pessoas ao nosso redor e a nós mesmos? Uma boa alternativa é a gente mesmo buscar os nossos direitos e exercê-los no nosso dia a dia e de forma ativa.

A lista abaixo traz algumas informações que poderão te ajudar a exercer os seus direitos de forma mais ativa e protagonista:

- **Fundar, Reabrir ou Participar do Grêmio de sua Escola:** já pensou em se juntar com outros jovens da sua escola e, juntos, pensarem nos problemas que vocês têm e como vocês podem resolvê-los?
A participação, reativação ou fundação de um Grêmio estudantil é uma forma de fazer isso! Os estudantes têm direito de se organizar e montar um grêmio na sua escola.
Bateu aquela curiosidade?
Então dá uma lidinha nesse site aqui:
www.ubes.org.br/gremios/

• **Conheça os seus Direitos, Conheça o ECA!** Você já ouviu falar do Estatuto da Criança e do Adolescente? Sabe para quê ele serve? Sabe como você pode utilizá-lo no seu dia a dia? O ECA é uma lei que prevê e garante todos os direitos das crianças e adolescentes do Brasil. No ECA constam as responsabilidades de toda a sociedade (família, escola, comunidade, etc.) na garantia desses direitos. Nesta cartilha, você encontra mais informações sobre o ECA (Opção 1 & Opção 2)

• **Núcleo de Cidadania:** fora da escola, muitas cidades têm um Núcleo de Cidadania dos Adolescentes, aberto para jovens e que ajuda a garantir os direitos dos jovens à participação ativa na sociedade. Para mais informações, acesse: www.unicef.org/brazil/nucleo-de-cidadania-de-adolescentes

• **Cultura e Lazer:** nada como participar de alguma atividade de cultura, arte ou esporte para se cuidar e prevenir problemas de saúde mental do seu coletivo de amigos. Consulte a agenda cultural da sua cidade, busque por pontos de cultura ou agentes locais. Aqui tem um mapa com algumas possibilidades no Brasil todo: www.mapas.cultura.gov.br
