# Supplementary material for: Cadê o Kauê? Co‐design and acceptability testing of a chat‐story aimed at enhancing youth participation in the promotion of mental health in Brazil
Source: J Child Psychol Psychiatry. 2024 Dec 20;66(5):697–715. doi: 10.1111/jcpp.14078 (PMC12018289; doi:10.1111/jcpp.14078)
Supplement: Supplementary file 2 — Appendix S2. Guide for school use. [file JCPP-66-697-s002.pdf]

CADÊ O KAUÊ?

Uma experiência para inspirar ações transformadoras em saúde mental

GUIA PARA APLICAÇÃO NA ESCOLA

Projeto Engajadamente

CADÊ O KAUÊ?

Uma experiência para inspirar ações transformadoras em saúde mental

GUIA PARA APLICAÇÃO NA ESCOLA

Projeto Engajadamente

Publicado em 2022 pelo Projeto "Engajdamente: O Papel Ativo dos Jovens na Promoção de Saúde Mental"

Esta publicação está disponível para acesso livre ao abrigo da licença Atribuição-Uso Não-Comercial-Partilha 3.0 IGO (CC-BY-NC-ND 3.0 IGO) (http://creativecommons.org/licenses/by-nc-nd/3.0/igo/).

Citação sugerida: Engajadamente (2022). "Cadê o Kauê?" Uma experiência para inspirar ações transformadoras em saúde mental: Guia para aplicação na escola. Brasília, DF.

Realização

UNIVERSITY OF OXFORD
UnB
Talk2U

Financiado por

OXFORD POPULATION HEALTH
The British Academy

SUMÁRIO

Apresentação 5

Parte 1: Fundamentos teóricos 6

Parte 2: A Ferramenta digital *Cadê o Kauê?* 9

Como usar a ferramenta digital em sala de aula? 13

Parte 3: A Roda de Conversa *Cadê o Kauê?* 14

Perguntas mobilizadoras 15

Parte 4: Sustentando a experiência no cotidiano da escola 17

Considerações finais 19

Colaboradores 20

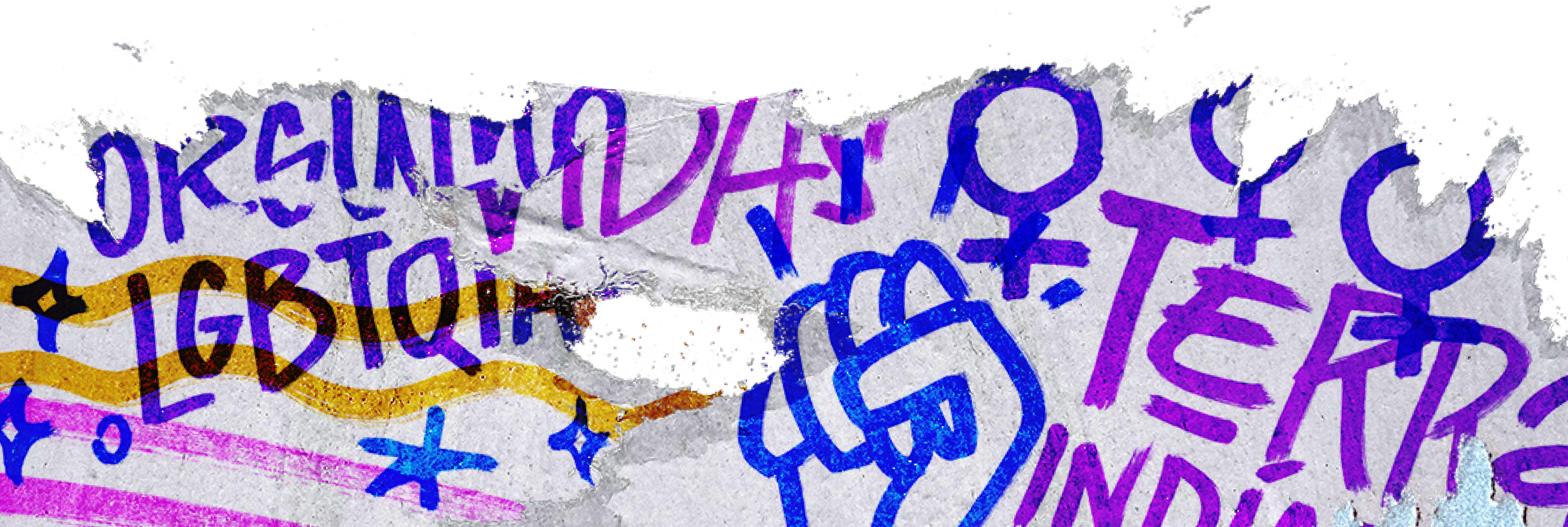

APRESENTAÇÃO

Até onde vai o poder de uma história contada na sua escola? A vida é, entre muitas coisas, um encadeamento de histórias, numa rede complexa que nos move, nos emociona, nos faz atores em cenários dos mais diversos. *Cadê o Kauê?* é uma história em formato de bate-papo online que aborda o poder do protagonismo juvenil para os desafios de saúde mental de jovens.

Nessa história, Kauê, um amigo fictício, acaba de experimentar uma reação emocional intensa na escola e, por isso, se esconde de todos. Na jornada em busca de Kauê, as estudantes e os estudantes que se engajam com a história refletem sobre as escolhas necessárias para encontrar o amigo que sumiu. À medida em que avançam e com o apoio de colegas e um professor, cada estudante descobre-se agente de mudança social na construção de uma escola mais acolhedora, que fomenta o apoio mútuo e ações transformadoras de jovens em prol da saúde mental.

A ferramenta foi pensada como um recurso para estimular conversas e reflexões com e entre estudantes sobre saúde mental e o papel de jovens enquanto agentes de mudança social. Contudo, a potência e a força transformadora da ferramenta está no envolvimento humano e na parceria da comunidade de aprendizagem, especialmente das professoras e dos professores.

Por isso, recomendamos que, depois de vivenciar a ferramenta digital, professores envolvam suas turmas de estudantes em uma roda de conversa para discutir e refletir sobre a experiência pensando a realidade da escola. A ideia é que essas conversas possam abordar oportunidades de ação, usando a ficção para estranhar a realidade e projetar futuros. A depender da qualidade de interlocução com e entre estudantes, diferentes resultados são percebidos. Assegurar espaços de escuta, o respeito a diferentes pontos de vista e apoiar que a voz jovem tenha influência são diferenciais importantes para que a roda contribua para processos de transformação social.

A experiência Cadê o Kauê? foi coproduzida por uma equipe composta por jovens, pesquisadores e roteiristas, em colaboração com professores e estudantes, cineastas e especialistas em tecnologia para apoiar a população de adolescentes no ensino médio de todas as regiões do Brasil. A ferramenta digital e a roda de conversa foram inspiradas nos resultados gerados por um mapeamento qualitativo das aspirações e barreiras ao protagonismo juvenil no apoio entre pares e na promoção da saúde e do bem-estar. Este mapeamento contou com 48 jovens entre 15-18 anos de todo o Brasil. Também foi conduzido um estudo quantitativo com 1891 jovens de mesma idade, que mapeou nacionalmente esferas de atuação jovem e necessidades de treinamento.

Este guia apresenta sugestões e instrumentos para a aplicação prática da ferramenta digital e da roda de conversa que formam a experiência Cadê o Kauê?. Agradecemos por construir o espaço de protagonismo de jovens em parceria pela saúde mental e por uma escola emancipatória e acolhedora.

Equipe Engajadamente

PARTE 1: FUNDAMENTOS TEÓRICOS

O QUE É SAÚDE MENTAL?

Quando se fala em “saúde mental”, muitas pessoas imediatamente pensam em “doença mental”. Entretanto, a saúde mental é definida como um estado de bem-estar completo[1], um atributo humano universal que pode envolver relações sociais positivas, percepção de pertencimento, de autonomia, senso de propósito, engajamento social, entre outros fatores. Experiências de sofrimento mental são diversas e variam de acordo com cada cultura, de pessoa para pessoa e até mesmo ao longo do tempo para o mesmo indivíduo. Isso sugere que os processos de saúde e doença mental não são conceitos binários, estar saudável é muito mais do que não estar doente. O sofrimento mental é melhor compreendido como um contínuo, desde sintomas leves e passageiros até dificuldades graves e incapacitantes[2].

A saúde mental é fruto de processos coletivos, sendo influenciada por determinantes sociais, tanto estruturais quanto proximais[3]. Dentre os determinantes sociais estruturais, têm-se as iniquidades de renda, raciais e de gênero, as normas culturais e o acesso à educação, por exemplo. Dentre os determinantes sociais proximais, tem-se a qualidade do ambiente familiar, as relações de amizade, o acesso à moradia, alimentação, escola e lazer, por exemplo. Portanto, as condições sociais, históricas, culturais, econômicas e políticas em que as pessoas vivem interagem, de modo complexo, com processos genéticos, psicológicos e neurodesenvolvimentais de cada um favorecendo ou fragilizando a saúde mental ao longo da vida (Figura 1)[4]. Mas a influência desses fatores não se dá “de cima para baixo”; o indivíduo é agente ativo na construção de suas experiências, ambiente e sociedade[5].

Desta forma, a promoção, a prevenção e o cuidado com a saúde mental deve ser multidimensional, considerando aspectos biopsicossociais. É preciso construir práticas que façam sentido desde o chão da escola, com as juventudes, para reconhecer, proteger e comunicar o que produz vida. Assim, as intervenções efetivas de educação em saúde mental requerem que as pessoas sejam vistas como participantes ativas no cuidado com a vida e não apenas espectadores do processo ou objeto de intervenções de especialidades e conhecimentos de terceiros. Isto envolve o reconhecimento e apropriação de um papel ativo, não somente em favorecer o cuidado de si, mas também de moldar iniciativas que afetem a todos.

---

[1] Organização Mundial de Saúde (OMS). Organização Pan-Americana de Saúde (OPAS). Relatório sobre a saúde no mundo: Saúde mental: Nova concepção, nova esperança. Brasília; 2001.

[2] Keyes, C. L. M. (2002). The mental health continuum: From languishing to flourishing in life. Journal of Health and Social Behavior, 43, 207–222.

[3] Viner, R. M., Ozer, E. M., Denny, S., Marmot, M., Resnick, M., Fatusi, A., & Currie, C. (2012). Adolescence and the social determinants of health. Lancet, 379, 1641–1652.

[4] Patel, V. et al., (2018). The Lancet Commission on Global Mental Health and Sustainable Development. The Lancet, 392, 10157.

[5] Bronfenbrenner, U. (1996). A Ecologia do desenvolvimento humano: Experimentos naturais e planejados. Porto Alegre, RS: Artmed.

	Fatores distais	Fatores proximais
**Demografia**	Diversidade comunitária<br>Igualdade de gênero	Idade<br>Raça e etnia<br>Gênero
**Economia**	Recessões econômicas<br>Desigualdade de renda	Renda / Dívida / Bens<br>Restrições financeiras<br>Pobreza relativa / Emprego<br>Segurança alimentar
**Vizinhança**	Infraestrutura<br>Escassez<br>Ambiente construído	Segurança e proteção<br>Estrutura habitacional<br>Superlotação<br>Recreação
**Ambiental**	Desastres naturais ou industriais<br>Guerra/conflito<br>Mudança climática<br>Migração forçada	Traumas<br>Violências
**Sociocultural**	Capital social comunitário<br>Estabilidade social<br>Cultura	Capital social individual<br>Participação social<br>Suporte social<br>Educação

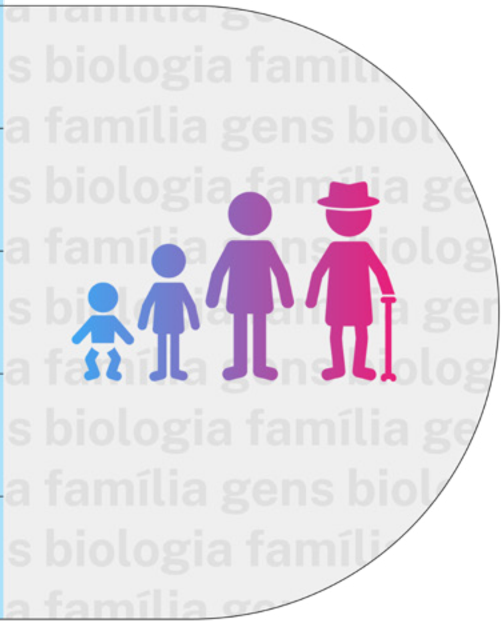

Figura 1. *Determinantes sociais da saúde mental global. Adaptado da Comissão Lancet de Saúde Mental Global e Desenvolvimento Sustentável. Tradução nossa.*

PROTAGONISMO JUVENIL NA SAÚDE MENTAL

"Saúde" é o tópico que mais motiva o engajamento cívico de jovens brasileiros[6]. Além disso, jovens tendem a escolher a escola como o lugar onde se reconhecem mais propensos a fazer alguma diferença no campo da saúde mental[7,8]. Uma escola transformadora deve dar aos adolescentes a chance de conversarem abertamente sobre os temas que os afetam e, a partir de reflexões articuladas do individual ao coletivo, encontrar soluções diante das barreiras percebidas. Ao favorecer o protagonismo juvenil em prol da saúde mental, a escola contribui para a formação de cidadãos autônomos, solidários e agentes de profundas transformações sociais.

Além da contribuição para a formação de pessoas cidadãs autodeterminadas e ativas em seus contextos sociais, a proposição de ações e ferramentas que coloquem jovens em um lugar de protagonismo nos assuntos e ações que dizem respeito à vida deles é também um direito previsto tanto pelo Estatuto da Criança e do Adolescente quanto por tratados internacionais como a Convenção Internacional dos Direitos da Criança.
Esses dois marcos legais se baseiam no *princípio dos melhores interesses da criança/adolecente*[9], o qual, de modo geral, estabelece que a criança/adolescente, em função da sua condição peculiar de desenvolvimento, deve ser uma prioridade de cuidado e atenção do Estado e da sociedade.

O QUE É PROTAGONISMO JUVENIL?

O protagonismo juvenil é a expressão, participação ou mobilização jovem para influenciar na tomada de decisão sobre questões que lhes dizem respeito direta ou indiretamente[10]. Não se trata de participação "de fachada", pois requer espaços seguros e audiência disposta a considerar a voz jovem e seu poder de influência sobre processos decisórios. Tampouco é "jogar a responsabilidade" sobre jovens; ao contrário, pressupõe a formação de parcerias intergeracionais em que todos envolvidos, adultos e jovens, co-construam soluções para os problemas que afligem a juventude

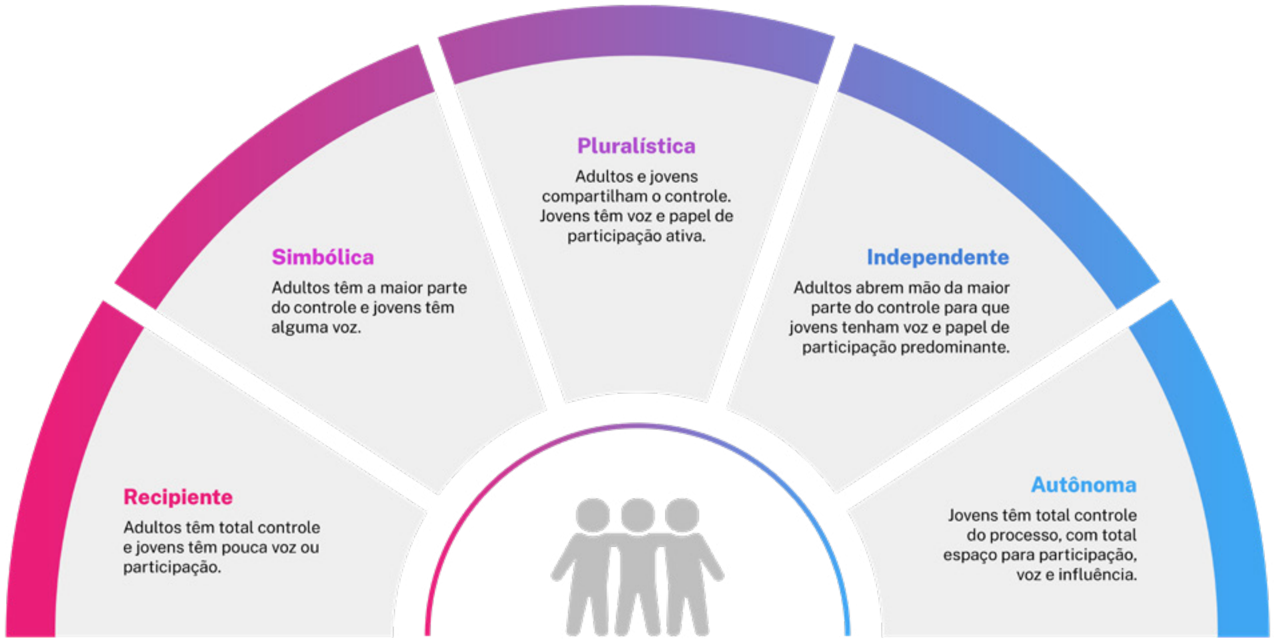

Figura 2. *Possibilidades do protagonismo juvenil.[11] Neste leque do espectro de participação da juventude, a centralidade do poder de condução de uma iniciativa se distribui entre, na ponta esquerda, o adulto liderando, na ponta direita, o jovem liderando. O meio sinaliza um poder compartilhado.*

[6] Anderson, M., & Jiang, J. (2018). Teens, social media & technology. Pew Research Center, 31, 2018.
[7] Boghossian, C. O., & Minayo, M. C.M. (2009). Revisão sistemática sobre juventude e participação nos últimos 10 anos. Saúde & Sociedade, 18, 411-423.
[8] Pavarini, G. et al. (2022). Agents of change for mental health: A survey of young people's aspirations for participation across five low-and middle-income countries. Journal of Adolescent Health.
[9] Mendes, J. A. A., & Ormerod, T. (2019). O princípio dos melhores interesses da criança: uma revisão integrativa de literatura em inglês e português. Psicologia em Estudo, 24.
[10] UNICEF (2020). Engaged and heard! Guidelines on adolescent participation and civic engagement. New York: United Nations Children's Fund.
[11] Wong, N.T., Zimmerman, M.A., & Parker, E.A. (2010). A Typology of Youth Participation and Empowerment for Child and Adolescent Health Promotion. American Journal of Community Psychology, 46, 100_114.

PARTE 2: A FERRAMENTA DIGITAL *CADÊ O KAUÊ?*

COMO FUNCIONA A FERRAMENTA?

Cadê o Kauê? é uma ferramenta que conta uma história em forma de troca de mensagens por meio de um bate papo online. É como conversar com uma amiga ou amigo no Messenger, Instagram ou Telegram mas as respostas são automatizadas. Essa interação pode dispor não só do envio de textos, mas também de áudios, vídeos e imagens. Uma vez engajados, as e os adolescentes saem do papel de mero espectador e assumem um papel ativo na história por meio das escolhas e reflexões que fazem.

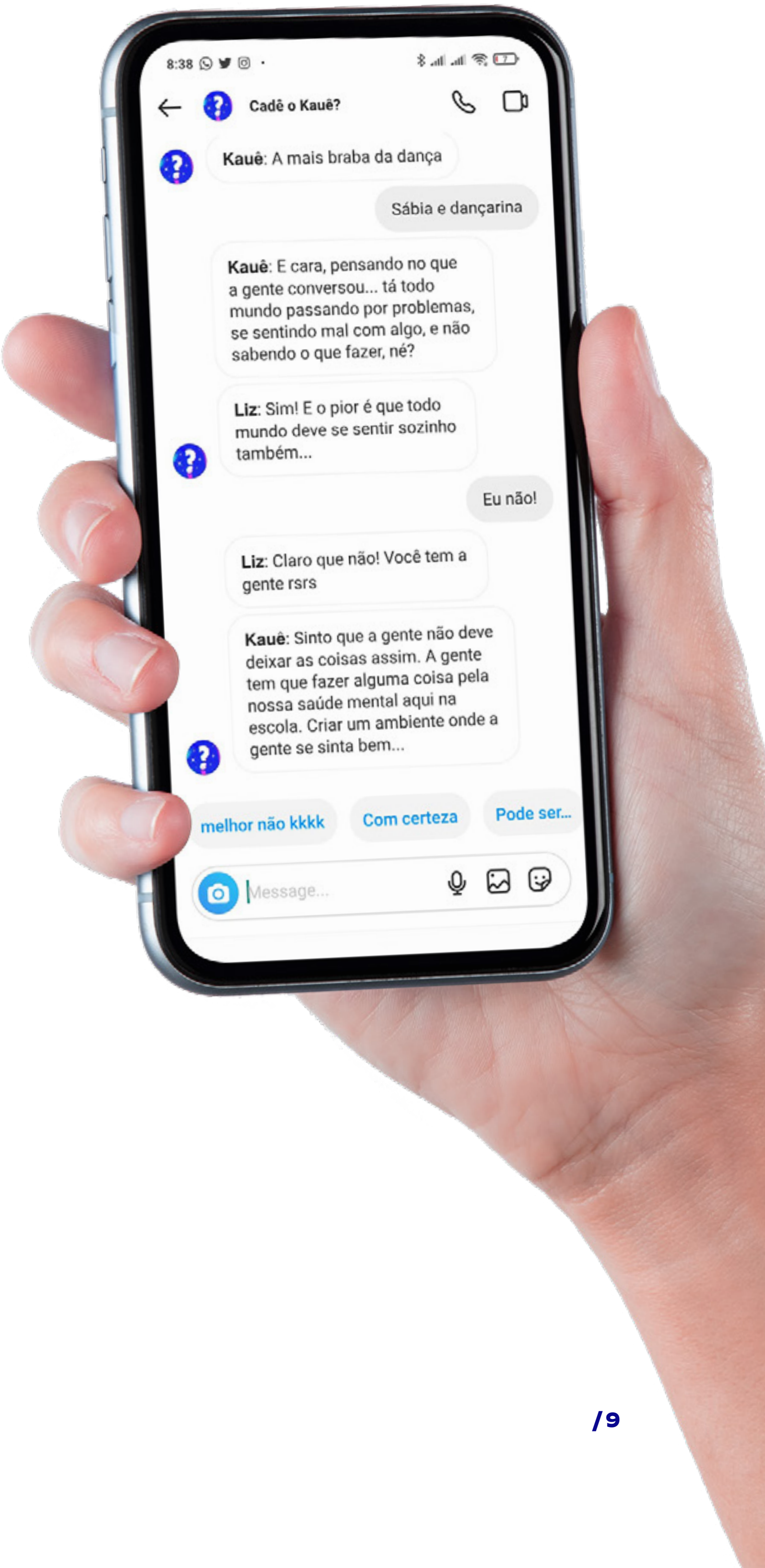

EM LINHAS GERAIS, COMO É A HISTÓRIA?

Imagine que um estudante chegue à escola e se depare com a seguinte situação: o melhor amigo, Kauê, desapareceu dentro da escola, no dia em que fariam a apresentação da peça teatral mais importante do ano. Kauê é personagem principal, e este sumiço também preocupa o professor Marcos, diretor da peça. A missão é encontrar Kauê com a ajuda de sua melhor amiga, Liz. No caminho, cada estudante encontra pistas do paradeiro do Kauê por meio de interações com outros personagens da história, que ajudam a entender o que estava acontecendo com o Kauê. Esse processo faz com que os estudantes tenham a oportunidade de apoiar outras amigas e amigos fictícios da história e também possam realizar ações transformadoras para uma escola mais sensível à saúde mental. Ao longo da experiência, são abordados temas como pressão escolar, pobreza, machismo, racismo, exposição digital, conflitos familiares, entre outros fatores que impactam a saúde mental.

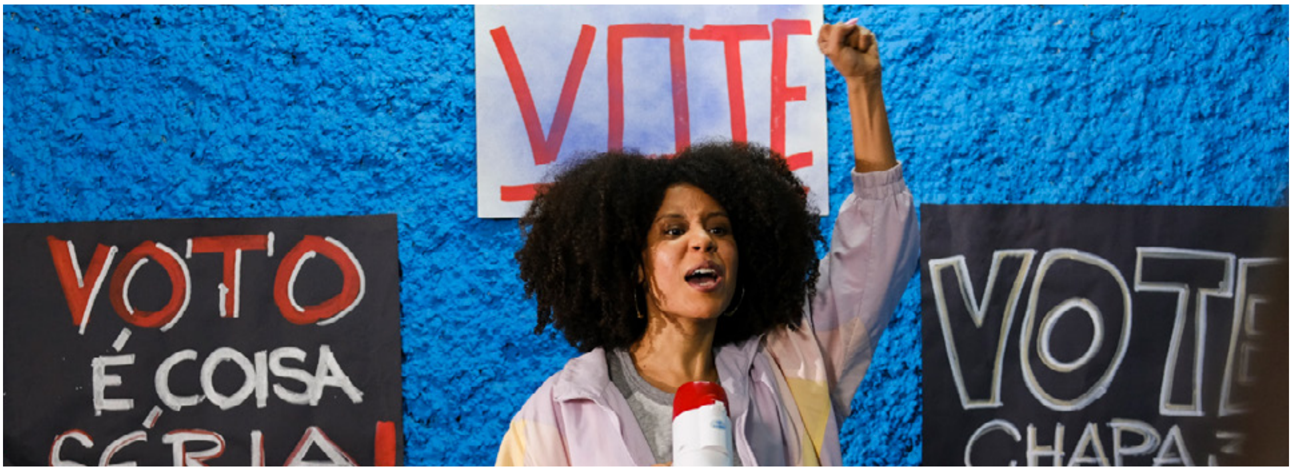

COMO A FERRAMENTA PODE INSPIRAR O PROTAGONISMO JUVENIL?

Durante conversas com as personagens, cada estudante entrará em contato com informações que podem ajudá-lo em seu papel de agente de mudança em prol da saúde mental, incluindo onde procurar ajuda, como criar um grêmio estudantil e fontes de apoio na comunidade.

À medida em que avançam na busca pelo Kauê, estudantes acessam “poções”, as quais são aprendizados que podem ser transpostos para a vida real. No total, são 13 poções (Tabela 1), que possibilitam compreender melhor como aprimorar, construir e fortalecer as habilidades para apoiar seus pares e transformar o ambiente ao redor, por exemplo organizando iniciativas que beneficiem o coletivo da sala de aula ou escola.

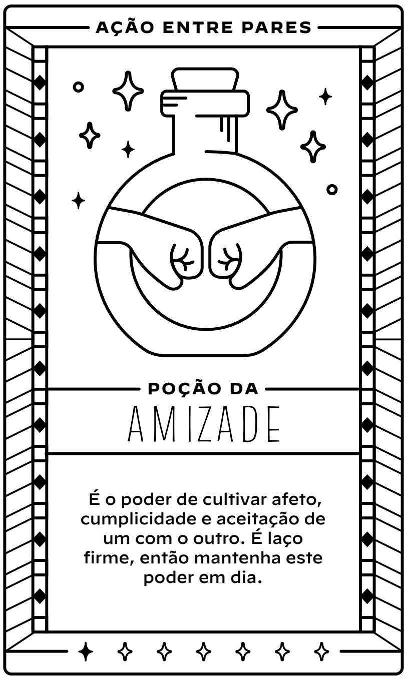

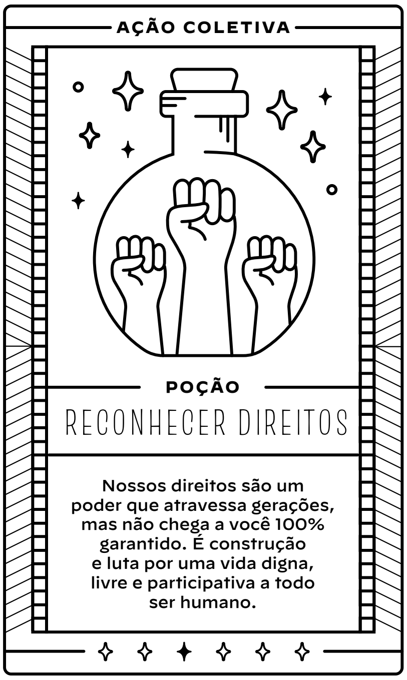

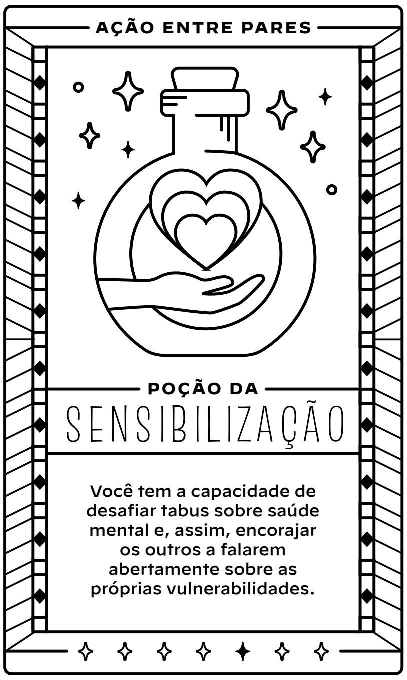

APOIO ENTRE PARES

DIMENSÕES /POÇÕES	DEFINIÇÕES
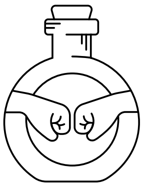 AMIZADE	Cultivar afeto, cumplicidade e aceitação mútua e evitar pressão e conflitos que revelam ausência de sintonia com o outro
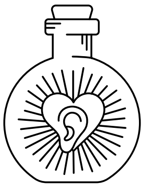 ESCUTA EMPÁTICA	Ouvir o outro sem julgamentos, compreender a sua perspectiva e validar seus sentimentos e/ou experiência
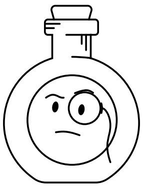 ATENÇÃO	Perceber mudanças de comportamento no outro, as quais possam revelar potenciais sinais de dificuldades de saúde mental
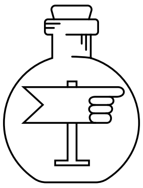 INDICAR APOIO	Informar os pares sobre fontes de apoio à saúde mental, tais como fontes de informação, adultos confiáveis e serviços especializados
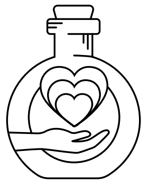 SENSIBILIZAÇÃO	Desafiar tabus sobre saúde mental e, assim, encorajar os outros a falarem abertamente sobre as próprias vulnerabilidades
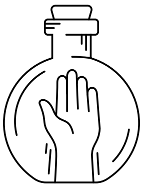 PEDIR AJUDA	Pedir orientação, informação ou apoio a fontes que capacitam jovens a proverem ajuda de forma mais segura e mais efetiva
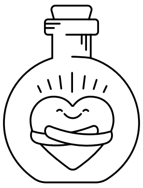 AUTOCUIDADO	Reconhecer os próprios sentimentos e respeitar os próprios limites antes, durante e após a oferta de ajuda para evitar sobrecarga ao ajudar

AÇÃO COLETIVA

DIMENSÕES /POÇÕES		DEFINIÇÕES
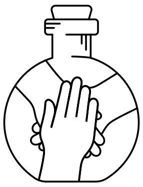	**PROBLEMAS COLETIVOS**	Identificar, não banalizar e entender melhor os problemas que afetam o coletivo, como os determinantes sociais da saúde mental
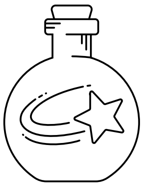	**SONHO**	Vislumbrar um futuro melhor e engajar-se nas mudanças necessárias para um mundo mais justo e acolhedor
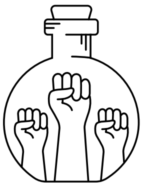	**RECONHECER DIREITOS**	Perceber-se merecedor de uma vida digna, livre e participativa, assim como todo e qualquer outro ser humano
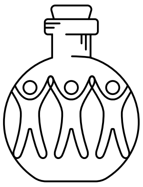	**UNIÃO**	Juntar-se a outras e outros jovens e torná-los aliados para realização de uma ação coletiva pelo bem-estar do grupo
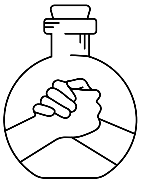	**PARCERIA**	Criar laços com pessoas ou entidades que inspiram, apoiam e orientam nas frentes de ação em prol da saúde mental
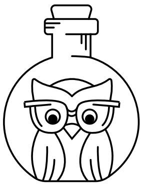	**DISCERNIMENTO**	Diferenciar fortalezas e riscos associados às várias formas de engajamento cívico pela saúde mental

Tabela 1. *Dimensões de participação juvenil centradas no apoio entre pares e ação coletiva.*

COMO USAR A FERRAMENTA DIGITAL EM SALA DE AULA?

A ferramenta digital leva aproximadamente 1h40m. Pode ser realizada em uma aula dupla ou como tarefa de casa.

Pode ser acessada através do telefone celular, computador ou tablet. A ferramenta foi pensada para uso individual, mas pode também ser realizada coletivamente.

Estima-se que, para uma turma de 30 estudantes, sejam necessários pelo menos 10 GB de dados para completar a experiência; verifique se a franquia de dados do plano de internet disponível para a escola é suficiente para o uso da ferramenta.

A história envolve áudio, então é desejável o uso de fones de ouvido quando a ferramenta for utilizada em sala de aula ou no laboratório de informática.

É possível acessar a ferramenta no site do Engajadamente (www.engajadamente.org) clique em **"Chat"** no canto direito da tela e depois em: "Continue as guest" ou "Continue como convidado". Ela também está disponível no recurso de envio de mensagens privadas do Instagram (envie "oi" para @cade_o_kaue).

Quando concluir a experiência, clique em "Terminar o bate-papo", assim estará pronto para a próxima pessoa.

Atenção nº1: Confira se a sua escola usa firewalls, bloqueios, contra o uso de mídias sociais. Neste caso, peça ajuda para desabilitar ou colocar em modo de "suspender firewalls" durante o uso da experiência.

Atenção nº2: Não utilize o modo anônimo do navegador. Ao usar o modo anônimo, os estudantes perdem o recurso de continuar onde pararam na história. No tempo de ir e voltar do banheiro tudo pode ter sido reiniciado.

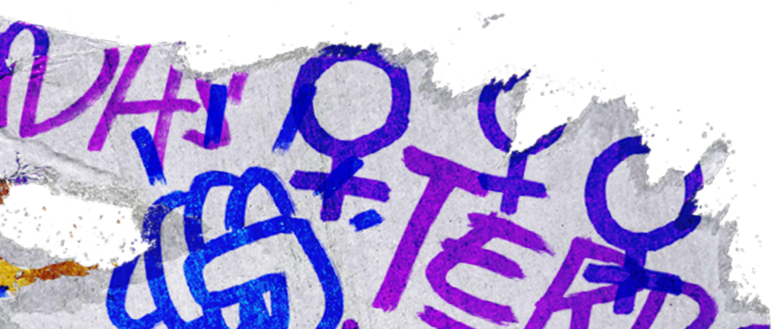

PARTE 3: A RODA DE CONVERSA *CADÊ O KAUÊ?*

Na aula subsequente à experiência, recomendamos que professores facilitem uma roda de conversa com a turma, a partir das habilidades que tiveram contato ao acessar as poções ao longo da experiência. A roda de conversa baseada na experiência tem o intuito de inspirar reflexões sobre a saúde mental entre jovens e o papel ativo deles, em parceria com a escola, para a promoção de saúde e bem-estar em suas relações sociais imediatas e em contextos sociais mais amplos, como a sala de aula, a escola e a sociedade.

CRIANDO UM AMBIENTE ACOLHEDOR

Muitas e muitos jovens nunca tiveram a chance de conversar sobre saúde mental e grande parte considera o assunto tabu ou tem uma ideia estereotipada sobre o tema. Por isso, é muito importante criar um ambiente acolhedor para que essa conversa aconteça, e que, idealmente, este espaço continue a existir transversalmente a todo o processo pedagógico.

É importante destacar que os estudantes têm co-responsabilidade na construção da sala de aula como um ambiente seguro, confiável e democrático. Vale a pena construir acordos e combinados a serem cumpridos coletivamente, por exemplo:

- Para que pratiquem a escuta ativa, respeitando a diversidade de pensamentos;
- Para que todas as pessoas possam falar (ou se abster) sem sofrer julgamentos, censuras ou silenciamentos;
- Para que haja uma distribuição justa dos turnos de fala, de modo que todas as pessoas tenham espaço para contribuir.

Por tratar de um tema sensível, alguns estudantes podem compartilhar experiências pessoais que indiquem necessidade de cuidado em saúde mental. É importante acordar com a turma que as vivências compartilhadas por quem estiver participando sejam mantidas em sigilo. A ferramenta digital dará às pessoas nelas engajadas acesso a um guia em PDF, onde há informações sobre diversos serviços de saúde mental, caso sintam necessidade de maior apoio.

É possível que sejam identificadas situações em que uma determinada pessoa precisará ser encaminhada para atendimento especializado ou ainda que alguma situação precisará ser reportada ao Conselho Tutelar. Nesses casos, é aconselhável conversar sobre o caso com o jovem e a equipe pedagógica da escola para decidirem o melhor manejo.

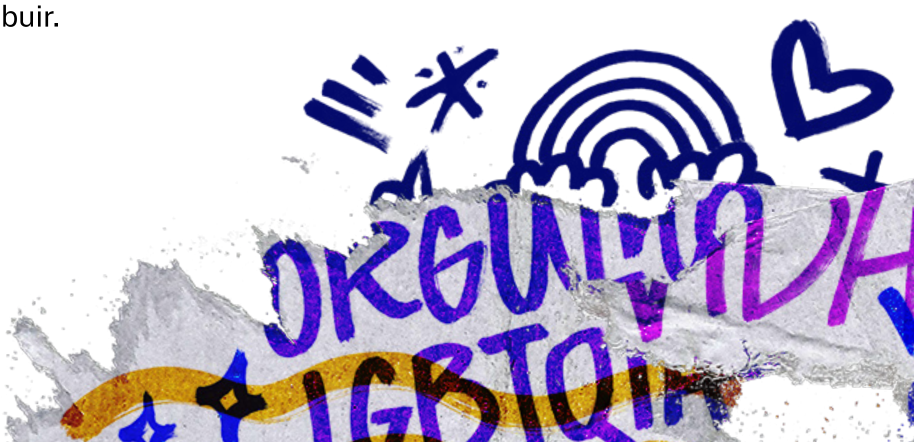

PERGUNTAS MOBILIZADORAS

É importante destacar que este guia é apenas um farol que pretende iluminar o trabalho docente, sem engessá-lo. Assim, sugerimos as seguintes perguntas mobilizadoras para a roda de conversa:

1

O que precisamos e podemos fazer para que ninguém da nossa turma, precise se esconder no banheiro, como fez o Kauê?

Como vocês acham que podemos nos ajudar mutuamente?

DICA: REFLITA SOBRE:

A) o que eu posso fazer enquanto amiga ou amigo e
B) o que nós podemos fazer enquanto turma?

2

Na história, o Kauê recebeu apoio, a peça foi reescrita, o banheiro foi reformado.

Há algo em específico que também podemos fazer para melhorar a saúde mental e o bem-estar da comunidade jovem da nossa sala de aula ou da nossa escola?

DICA: REFLITA SOBRE:

Não haver uma resposta certa ou errada. Encorajamos sugestões em todos os níveis, as quais possam apoiar de forma direta e indiretamente a saúde mental, que se alinhem com o que as e os estudantes consideram importante para si e para a comunidade.

3

Quais oportunidades e barreiras a nossa sala de aula e escola trazem para o protagonismo juvenil?

Com quem e com o que podemos contar para realizar a(s) ação(ões) sugeridas por vocês na nossa sala de aula e/ou escola?

DICA: Você pode sugerir a construção de um plano com três pontos:

A) o que fazer?, **B)** quando fazer?, e **C)** com quem? (ex.: grêmio estudantil) ou com o quê? (ex.: nossos talentos) poderemos contar.

Recomendamos que professores acompanhem cada estudante na implementação das propostas levantadas, viabilizando as condições para tal, em articulação com outros atores na escola, como o grêmio estudantil, a equipe pedagógica e, eventualmente, parceiros externos à escola. Estudantes, em parceria com professores e demais aliados, poderão construir, pouco a pouco, condições para influenciar o ambiente da sala de aula e, a depender da integração entre esta experiência e o projeto pedagógico da escola, o ambiente de aprendizagem **cultivado na escola** (ver Figura 3).

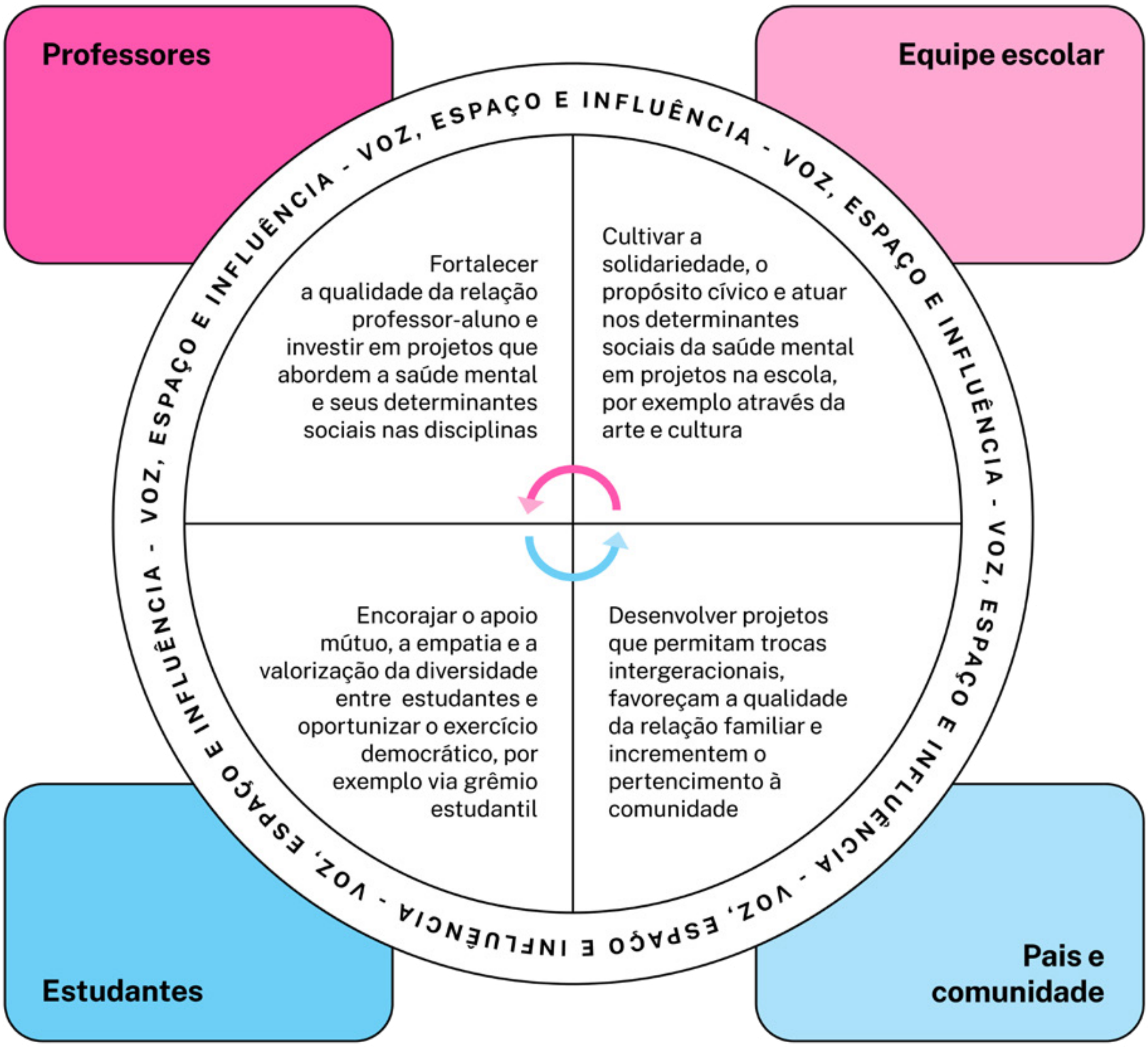

Figura 3. *Oportunidades para promoção do protagonismo juvenil em prol da saúde mental na escola.*

Nota: Esta figura trata das atribuições e papéis dos agentes que atuam no âmbito da escola e da comunidade escolar. É importante lembrar que a promoção da saúde mental na escola não depende apenas da escola e dos que compõem a comunidade escolar, mas do fomento de políticas públicas.

PARTE 4: SUSTENTANDO A EXPERIÊNCIA NO COTIDIANO DA ESCOLA

A ferramenta digital e a roda de conversa são contribuições a mais ao trabalho da unidade de ensino para sensibilizar para modos de relação na escola que oportunizem a ajuda entre pares e a ação coletiva para a promoção da saúde mental. Nesse sentido, é interessante que a realização da experiência *Cadê o Kauê?* seja integrada a outros projetos em andamento na escola, e que outros membros da equipe da escola, bem como coordenadoras(es) pedagógicos, mães, pais e outros membros da comunidade escolar possam contribuir para as ações realizadas.

O uso da ferramenta digital e a roda de conversa podem e devem ser estimuladoras de novas atividades no cotidiano das relações de ensino-aprendizagem que fortaleçam práticas pedagógicas favoráveis ao protagonismo juvenil em prol da saúde mental e dos direitos das crianças e adolescentes na escola.

Cabe lembrar que os pressupostos teóricos que subsidiaram a elaboração de *Cadê o Kauê?* estão em profundo diálogo com os objetivos de ensino propostos pelas Diretrizes Nacionais de Educação em Direitos Humanos e contribuem para se alcançar o ambiente da escola acolhedora descrita na Base Nacional Comum Curricular (BNCC, 2017, pp. 466-467).

SEGUNDO A BNCC, A ESCOLA DEVE FAVORECER QUE AS E OS ESTUDANTES:

- Se (re)conheçam e possam lidar melhor com seu corpo, seus sentimentos, suas emoções e suas relações interpessoais, fazendo-se respeitar e respeitando os demais;
- Combatam estereótipos, discriminações de qualquer natureza e violações de direitos de pessoas ou grupos sociais, favorecendo o convívio com a diferença;
- Valorizem sua participação política e social e a dos outros, respeitando as liberdades civis garantidas no estado democrático de direito;
- Construam projetos pessoais e coletivos baseados na liberdade, na justiça social, na solidariedade, na cooperação e na sustentabilidade;
- Possam ter garantido o seu protagonismo no processo de aprendizagem e no desenvolvimento de suas capacidades de abstração, reflexão, interpretação, proposição e ação, essenciais à sua autonomia pessoal, profissional, intelectual e política;
- Tenham seu papel social, para além de sua condição de estudante, valorizado, e que os processos de construção de identidade e projeto de vida sejam qualificados.

REFLEXÕES PARA PROFESSORES E EQUIPE PEDAGÓGICA

Para que a experiência *Cadê o Kauê?* contribua para o alcance dos objetivos de ensino previstos na BNCC, convém refletir, avaliar e discutir com toda a equipe pedagógica em que extensão a escola tem oferecido um ambiente seguro e propício para o protagonismo juvenil. Algumas questões podem nortear discussões em equipe:

- Como a qualidade da relação entre professores e estudantes tem facilitado ou dificultado o protagonismo juvenil, conforme a percepção de cada docente?
- Como as perspectivas de estudantes têm sido incorporada nas decisões que afetam o cotidiano da escola?
- Em que medida estudantes têm proposições consultadas e consideradas no processo decisório acerca de questões de seu interesse na escola?
- Quais espaços têm sido previstos no planejamento político-pedagógico da escola para que estudantes se engajem em ações solidárias em relação aos seus pares e na sua comunidade?
- Quais oportunidades e barreiras o estilo de gestão, cultura e clima escolar oferecem para o protagonismo juvenil?

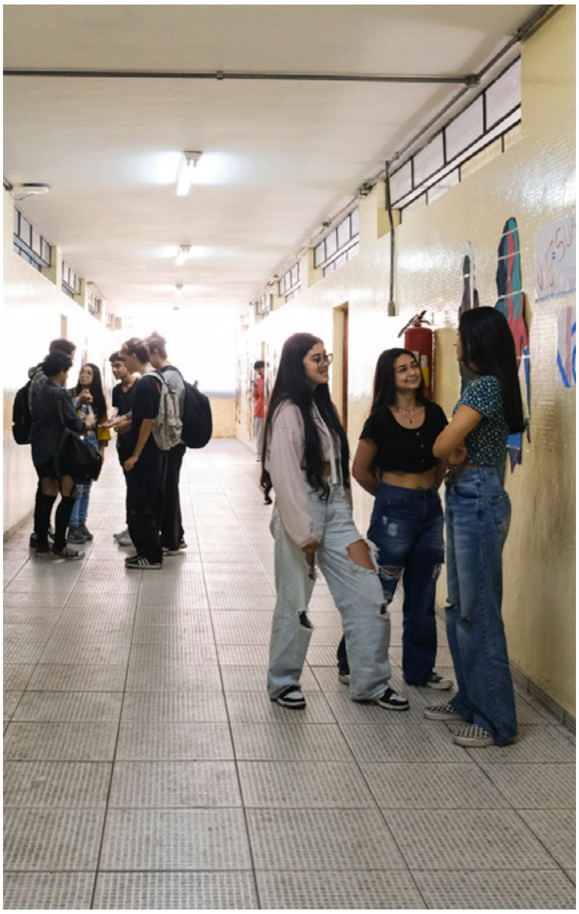

CONSIDERAÇÕES FINAIS

Cremos que há múltiplas maneiras pelas quais *Cadê o Kauê?* pode ser utilizado na escola. Apostamos na criatividade e na autonomia das professoras e dos professores de todo o Brasil para maximizar os potenciais benefícios da ferramenta e da roda de conversa na sua prática pedagógica e na sua escola. A ferramenta é disponibilizada de forma gratuita. Sugerimos dar continuidade à nossa jornada através da **hashtag #cadeokaue** no Instagram, postando dinâmicas, vivências, questionamentos, ações transformadoras, para que possamos continuar aprendendo em conjunto a construir uma escola mais democrática e solidária.

Projeto Engajadamente: @engajada.mente

Cadê o Kauê: @cade_o_kaue

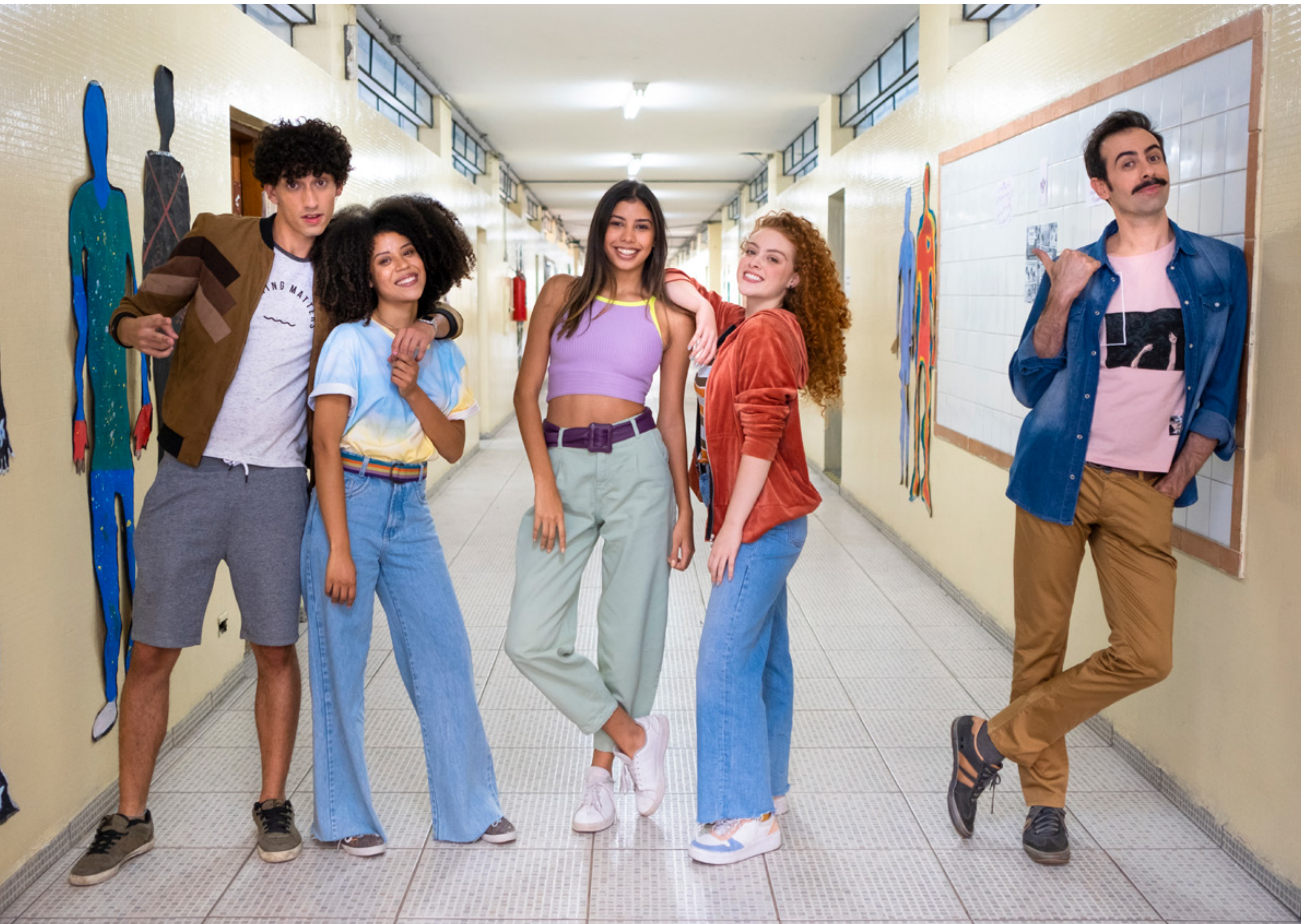

COLABORADORES

Cadê o Kauê? é fruto de uma colaboração entre pesquisadoras e pesquisadores das Universidades de Oxford e Brasília, um comitê colaborativo de jovens e a empresa de tecnologia Talk2U, em constante troca de experiências com membros da comunidade escolar, artistas e especialistas em políticas públicas.

Co-design da experiência *Cadê o Kauê?*

Equipe de pesquisadores
Dra. Gabriela Pavarini, Universidade de Oxford
Profa. Dra. Sheila Giardini Murta, Universidade de Brasília
Dr. Josimar A. de Alcântara Mendes, Universidade de Brasília
Dr. Felipe Rodrigues Siston, Universidade de Brasília

Comitê Colaborativo de Jovens
Brenda Thallys Rocha Seabra
Julyana Alves Ferreira
Rafael Ribeiro Alves de Souza
Rafaela de Oliveira da Cunha
Victor Hugo de Lima Santos

Talk2U
Nico Ferrario, Talk2U
Iasmim Coni, Talk2U
Bia Matera, Talk2U

Consultora em intervenções

Ana Luiza Savi, Talk2U

Apoio acadêmico

Profa. Dra. Ilina Singh, Universidade de Oxford

Produção técnica da ferramenta digital

Lucila Suarez Battan, Talk2U
Lucca Rosa Pereira, Talk2U

Produção audiovisual da ferramenta digital

Morena Flor
Orgnx_Filmes
Amparo Guindón
Sômolo Salvador

Consultoras em políticas públicas

Profa. Dra. Silvia Lordello, Universidade de Brasília
Profa. Dra. Larissa Polejack, Universidade de Brasília

Consultores em educação

Profa. Gina Vieira Ponte, professora aposentada da Secretaria de Estado de Educação do Distrito Federal.
Profa. Ana Paula Santiago S. Andrade, Coordenadora pedagógica no IFB - Campus Recanto das Emas (DF)
Profa. Claudia Cavalcante de Carvalho Weber, Pedagoga no Centro de Ensino Médio Setor Oeste (DF)
Profa. Fernanda Bernardes Silveira Costa, Centro de Ensino Médio 04 Sobradinho 2 (DF)
Profa. Gabriela Staerke de Rezende, Psicóloga no Centro de Ensino Médio Paulo Freire (DF)
Profa. Janete Araújo da Silva, Professora no CEL - Centro Educacional do Lago Sul (DF)
Prof. Jonas de Oliveira Bertucci, Professor no Instituto Federal de Brasília, Campus São Sebastião (DF)
Profa. Maria Salete Guerra Maranhão Bezerra, Equipe SOE do Centro de Ensino Médio Paulo Freire (DF)
Mikael Silva Rocha, Psicólogo no Centro de Ensino Médio 02 de Brazlândia (DF)
Prof. Rodrigo Araújo Magalhães, Equipe pedagógica da Regional de Ensino de Brazlândia (DF)
Profa. Rosimeire Aguiar Pereira Lopes, Centro de Ensino Médio 09 de Ceilândia e no Centro Educacional 11 de Ceilândia (DF)
Prof. Thiago Freire, Secretaria de Estado de Educação do Distrito Federal.

Consultores jovens

Alice Christovam Constantino, 19 anos, Faculdade de Tecnologia de Praia Grande, Mongaguá (SP)
Alicia Mendes, 17 anos, Instituto de Educação Carmela Dutra, Rio de Janeiro (RJ)
Ana Clara Freitas Ferreira, 16 anos, Centro de Ensino Médio 04, Sobradinho 2, Brasília (DF)
Ana Vitória Oliveira da Silva, 17 anos, Colégio Santa Mônica, Rio de Janeiro (RJ)
Brenda lorrane Ferreira Feitosa, 19 anos, Centro de Ensino Médio 04, Sobradinho 2, Brasília (DF)
Ester de Souza Alves de Souza, 17 anos, Escola de Educação Básica Professora Gracinda Augusta Machado, Imbituba (SC)
Emerson Gomes dos Santos, 16, Escola Padre José Bráulio Sousa Ayres, São Luís (MA)
Hevellyn Sofia Elvino Pinheiro, Unidade Escolar Severiano Souza, Teresina (PI)
Iasmyn Brito dos Santos, 18 anos, Universidade Estadual de Goiás, Formosa (GO)
Isis Santos Goulart, 16 anos, E.E.E.M Roque Gonzáles, Porto Alegre (RS)
Jamili Maine Lima Campos, 15 anos, Colégio Estadual Maria Montessori, Curitiba (PR)
Maria Layza Paixão, 17 anos, Escola Estadual de Pau da Lima (formada), Salvador (BA)
Milena Sousa, 17 anos, Colégio Estadual Raimundo Neiva de Carvalho (formada), Palmeiras do Tocantins (TO)
Mutula Manuel Simão João, 16 anos, Escola Estadual Ottilia Homero da Silva, Pinhais (PR)
Natália Samuel Pitcella Borba, 18 anos, Escola Técnica Estadual de Itanhaém, Itanhaém (SP)
Raissa Barbosa, 16 anos, Instituto Federal de Alagoas, Major Isidoro (AL).
Raissa Amarante, 18 anos, Instituto Federal de Brasília, Brasília (DF)
Yasmin Bilek, 17 anos, Setor de Educação Profissional e Tecnológica da Universidade Federal do Paraná, Curitiba (PR)
Yasmin Flores da Silva, 16 anos, Escola Barbosa Rodrigues, Gravataí (RS)

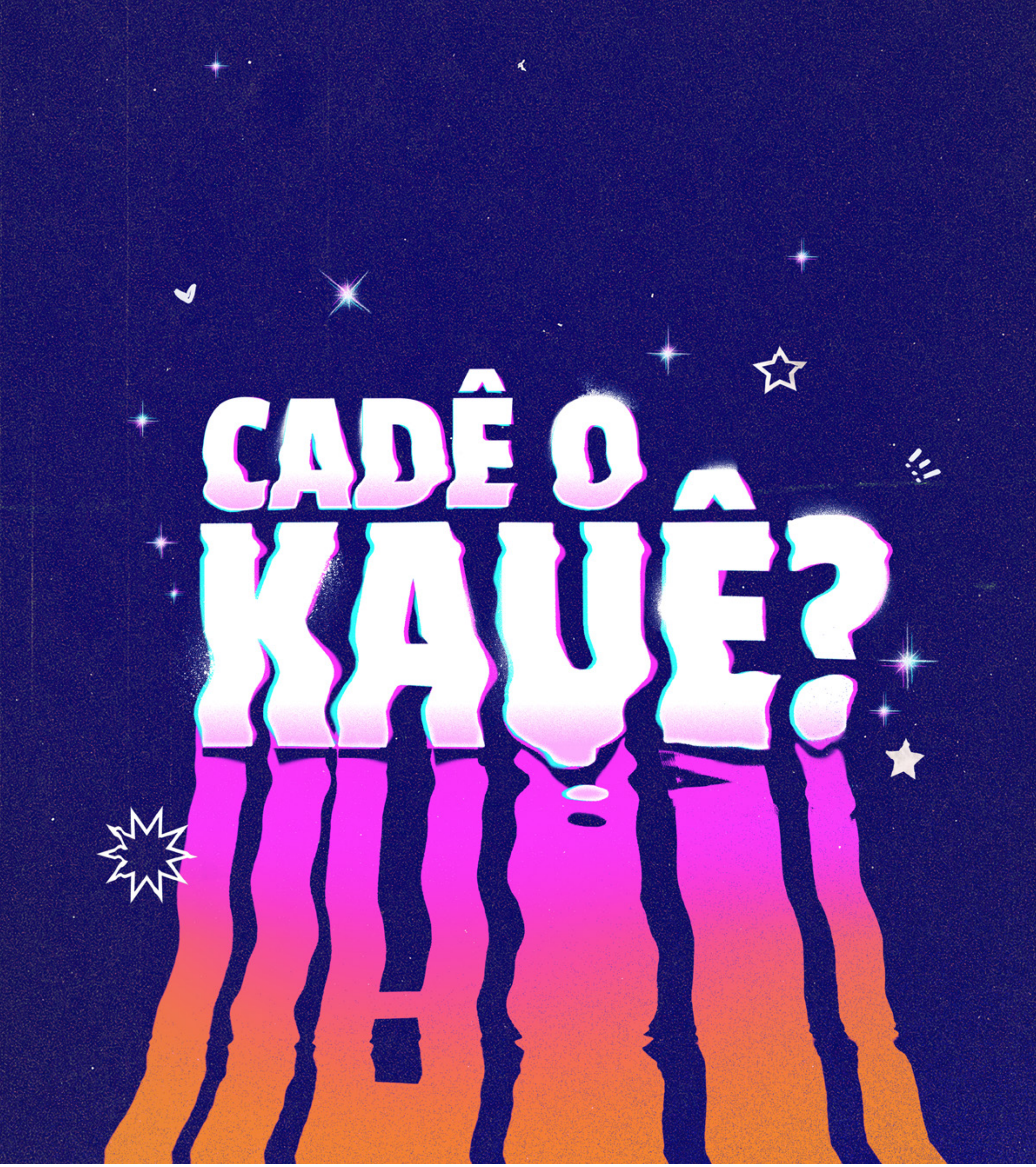

Realização

UNIVERSITY OF OXFORD

UnB

Talk2U

Financiado por

OXFORD POPULATION HEALTH

The British Academy
