# Supplementary material for: Cadê o Kauê? Co‐design and acceptability testing of a chat‐story aimed at enhancing youth participation in the promotion of mental health in Brazil
Source: J Child Psychol Psychiatry. 2024 Dec 20;66(5):697–715. doi: 10.1111/jcpp.14078 (PMC12018289; doi:10.1111/jcpp.14078)
Supplement: Supplementary file 3 — Appendix S3. User testing session 1 additional survey results. [file JCPP-66-697-s001.pdf]

# Supplementary 3. Results from user-testing session 1

Cadê o Kauê? Co-design and acceptability testing of a chat-story aimed at enhancing youth participation in the promotion of mental health in Brazil

Gabriela Pavarini, Sheila Giardini Murta, Josimar Antônio de Alcântara Mendes, Felipe Rodrigues Siston, Rafa Ribeiro Alves de Souza, Rafaela de Oliveira da Cunha, Julyana Alves Ferreira, Victor Hugo de Lima Santos, Brenda Thallys Rocha Seabra, Talk2U, Ilina Singh

*Address for correspondence: gabriela.pavarini@ethox.ox.ac.uk

| **Question** | **Results** |
|---|---|
| Has the game taken too long to begin? | No (n=9)<br>Yes (n=4)<br>It doesn’t matter to me (n=2) |
| What did you think of the information on the potion cards? | Too long (n=1)<br>A little long (n=8)<br>I haven’t noticed (n=1)<br>Right amount of info (n=4)<br>I didn’t want them anymore (n=1) |
| At any point, did you feel like you wanted to disagree with Duda? | Yes (n=10)<br>Sometimes (n=5)<br>No (n=0) |
| How much does the profile you received reflect who you are? How much does it make sense to you? (out of 10) | *M* = 8.00 (*SD* = 1.60) |
| How easy was it to navigate through the experience? (out of 10) | *M* = 8.67 (*SD* = 1.45) |
| How much did you enjoy the perspective of taking on a character’s role in this experience? (out of 10) | *M* = 8.87 (*SD* = 1.77) |
| What do you think about the duration of this learning experience? | Too long (n=4)<br>A little long (n=6)<br>Okay (n=5)<br>A little short (n=0)<br>Too short (n=0) |
| Did you have fun? | No, I was bored (n=1)<br>At first, then I got bored (n=3)<br>I wouldn't say I had fun (n=1)<br>Yes, I enjoyed the experience (n=9)<br>Yes, definitely, I loved it (n=1) |
| What did you enjoy the most about the experience? [Open field] | **Coded categories:**<br>User-experience (‘The interactivity, that was incredible’) (n=4)<br>Storyline (‘How the story developed’) (n=2)<br>Leanings (‘The help from authorities and exposure to the NGOs' help site) (n=2)<br>Everything (n=4)<br>Nothing (n=2)<br>Missing (n=1) |

| Question | Results |
|---|---|
| What do you think needs to be improved in the experience? [Open field] | **Coded categories:**<br>Length (n=5)<br>User experience elements ('more response options') (n=4)<br>No need to improve/Nothing (n=3)<br>Don't know (n=1)<br>Missing (n=2) |
| Did any part of the experience make you uncomfortable and/or touch on sensitive subjects? | Yes (n=7)<br>No (n=8) |
| Did you feel that the learning experience is a safe space to talk about mental health? | Yes (n=13)<br>No (n=2) |
| After this experience, what have you learned about mental health? [Open field] | **Coded categories**<br>Generic positive ('Many things') (n=6)<br>Importance of caring for mental health ('That we should take care of it') (n=2)<br>Importance of sharing ('We need to share our insecurities and difficulties') (n=3)<br>Importance of getting help ('That outside help is really necessary') (n=1)<br>Nothing new (n=2)<br>Missing (n=1) |
| Did the videos, audios, and images arrive quickly and in the correct order? | It seems so (n=9)<br>I don't know (n=2)<br>Sometimes it seemed to take a while (n=3)<br>Sometimes the order didn't make sense to me (n=0)<br>No, they seemed out of order (n=0)<br>Missing (n=1) |
| Did you encounter any bugs? | No (n=11)<br>Yes (n=3)<br>Missing (n=1) |
| How likely are you to recommend this learning experience to a friend? (out of 10) | *M*=8.21 (*SD*=2.00) |
